# Functional Mapping of Quantitative Trait Loci (QTLs) Associated With Plant Performance in a Wheat MAGIC Mapping Population

**DOI:** 10.3389/fpls.2018.00887

**Published:** 2018-07-09

**Authors:** Anyela V. Camargo, Ian Mackay, Richard Mott, Jiwan Han, John H. Doonan, Karen Askew, Fiona Corke, Kevin Williams, Alison R. Bentley

**Affiliations:** ^1^The John Bingham Laboratory, National Institute of Agricultural Botany, Cambridge, United Kingdom; ^2^Division of Bioscience, Genetics Institute, University College London, London, United Kingdom; ^3^National Plant Phenomics Centre, Institute of Biological Environmental and Rural Sciences, Aberystwyth University, Aberystwyth, United Kingdom

**Keywords:** wheat, senescence, data science, phenology, phenotyping, MAGIC

## Abstract

In crop genetic studies, the mapping of longitudinal data describing the spatio-temporal nature of agronomic traits can elucidate the factors influencing their formation and development. Here, we combine the mapping power and precision of a MAGIC wheat population with robust computational methods to track the spatio- temporal dynamics of traits associated with wheat performance. NIAB MAGIC lines were phenotyped throughout their lifecycle under smart house conditions. Growth models were fitted to the data describing growth trajectories of plant area, height, water use and senescence and fitted parameters were mapped as quantitative traits. Trait data from single time points were also mapped to determine when and how markers became and ceased to be significant. Assessment of temporal dynamics allowed the identification of marker-trait associations and tracking of trait development against the genetic contribution of key markers. We establish a data-driven approach for understanding complex agronomic traits and accelerate research in plant breeding.

## Introduction

In crop genetics, the formation of dynamic biological traits such as height, size and color are usually governed by multiple temporal and spatial factors. Their genetic variation can be attributed to the collective response of multiple small effects associated to those dynamic traits (Anderegg, 2015). Thus, some genes may control trait development at a given plant developmental stage, others may alter, or control rates of change, transitions between consecutive stages (Yang and Xu, 2007). These changes can be due to different genes that turn on or off at various times. In other words, a dynamic trait is, in part, governed by genes whose effects change with time. When a trait is measured over many developmental stages, e.g., plant area, it reveals the dynamic expression of the underlying genes associated with the trait (Wu et al., 2011). These might reveal critical aspects of vulnerability and response to biotic and abiotic stresses, and thereby predict the effects of climate change on these traits (Soudzilovskaia et al., 2013).

Up until recently, genetic mapping of traits such height, area or senescence was done on a single data point, representing the value of the trait at a given stage. This approach, although practical, overlooked many of the factors that defined the process of formation and development of the trait. When breeding for elite varieties, it is widely accepted that the timing of key developmental transitions is associated with crop performance. In wheat for example, the transition from vegetative to reproductive growth can have major effects on biomass accumulation and harvest index. Therefore, understanding the extent at which dynamics traits develop in time, an in space, may be useful in breeding for higher yield and stress adaptation.

Advances in phenotyping technology, as well as the development of methods for the analysis of longitudinal data have made possible the mapping of quantitative trait loci (QTLs) underlying the dynamics of a developmental trait. For example, Yang et al. (2006) fitted longitudinal data from four traits to a polynomial growth trajectory and used interval-mapping to map growth parameters to the genome. Yang and Xu (2007) also fitted growth trajectories to Legendre polynomials but used a Bayesian shrinkage model for multiple QTL mapping of the curve parameters. Polynomial models force growth to follow a smooth curve, potentially of great complexity. However, polynomial functions tend to make spurious predictions, their polynomial order is difficult to determine and their model parameters difficult to interpret (Paine et al., 2012; Xiong et al., 2011) used a general functional regression approach to fit mouse behavioral data. Li and Sillanpää (2013) used a Bayesian non-parametric multiple-loci procedure. The method uses the Bayesian P-splines with (non-parametric) B-spline bases to specify the form of the QTL trajectory and a random walk prior to determining the curve's degree of smoothness. Al-Tamimi et al. (2016) used cubic smoothing splines to estimate plant growth and transpiration in a rice population over 13 days. Although they fitted a growth model, they did not map the curve's fitted parameters but instead individual time ranges.

In this study we analyzed phenotypic profiles derived from the daily screening of a large, eight-founder “NIAB elite MAGIC” wheat population to evaluate the genetic factors underlying the temporal changes in key traits associated with wheat performance such as plant height and water use. Growth curve models were fitted to plant area, height, water use and senescence over time and the fitted parameters of the growth trajectories were mapped to the wheat genome. Single time points were also individually mapped to the genomes and results from both analytic approaches compared. We demonstrate that both methods provide key insights that could not be captured otherwise. The growth curve approach identifies crucial markers through the crop growth time line and the single time point approach gives an indication of when and how those markers become and then cease to be significant.

## Materials and methods

### Plant material

Phenotypes extracted from a subset of the NIAB Elite eight-founder MAGIC population described in Mackay et al. (2014) were evaluated in this study. The complete population consists of approximately 1,000 recombinant inbred lines (RILs) generated from three cycles of recombination between eight elite United Kingdom wheat varieties followed by five rounds of selfing to derive RILs. More information about the population including complete pedigrees, genotypes and existing phenotyping data can be found at www.niab.com/MAGIC.

### Glasshouse cultivation

As described in Camargo et al. (2016), plants were assessed between mid-January 2015 and mid-April 2015 in a Smart house at The National Plant Phenomics Centre facilities in Aberystwyth, UK. MAGIC parents together with 208 RILs (Camargo et al., 2016) were sown on 20 Oct, 2014 under well-watered conditions, with two replicates per genotype. Two seeds were sown in 6 cm pots of Levington F2 compost. After germination [11 Oct, 2014, approximately 11 days after sowing (DAS)] the seedlings were thinned to one per pot and transferred to a controlled environment room for vernalization (5°C, 16-h day length) for 9 weeks. Following vernalization plants were transplanted to 15 × 15 × 20 cm pots of F2 compost and were transferred to the Smart house. Each pot was placed into a cart on a conveyor system to allow for automatic and regular phenotyping. Pots were weighed and watered automatically daily to 75% gravimetric soil water content. Growth conditions were set to 14-h day-length using 600 W sodium lamps to supplement natural lighting. Temperature was set to 18°C day, and 15°C night.

### Phenotyping

Daily imaging commenced when the plants were transferred to the conveyor belt following vernalization using a LemnaTec 3D Scanalyzer (LemnaTec, GmbH, Wuerselen, Germany) for image acquisition. Four RGB pictures (2,056 × 2,454 pixels) were taken of each plant, one top view and three side view with a 45° horizontal rotation. All manually acquired and digitally derived traits and their abbreviations are provided in Table 1.

**Table 1:** Plant trait descriptions.

**Abbreviation in paper**	**Trait**	**Trait description**	**Unit**
Area	Area	Plant area in mm^2^	mm^2^
Height	Height	Plant height	mm^2^
TYP.Area	Senescence	Total yellow area (senescence)	mm^2^
water_amount	Water Use	Water amount	ml
A	A	Minimum asymptote (Equation 2)	
*C*	C	x value at the inflection point of the curve (Equation 2)	
*B*	B	Slope factor (Equation 2)	
*D*	D	Maximum value that can be obtained (Equation 2)	
*μ*	*μ*	Curve's mean (Equation 12)	
*λ*	*λ*	Curve's amplitude (Equation 12)	
*σ*	*σ*	Curve's standard deviation (Equation 12)	
*S*	*S*	Bimodal separation (Equation 12)	

### Image processing

**Figure 1:**
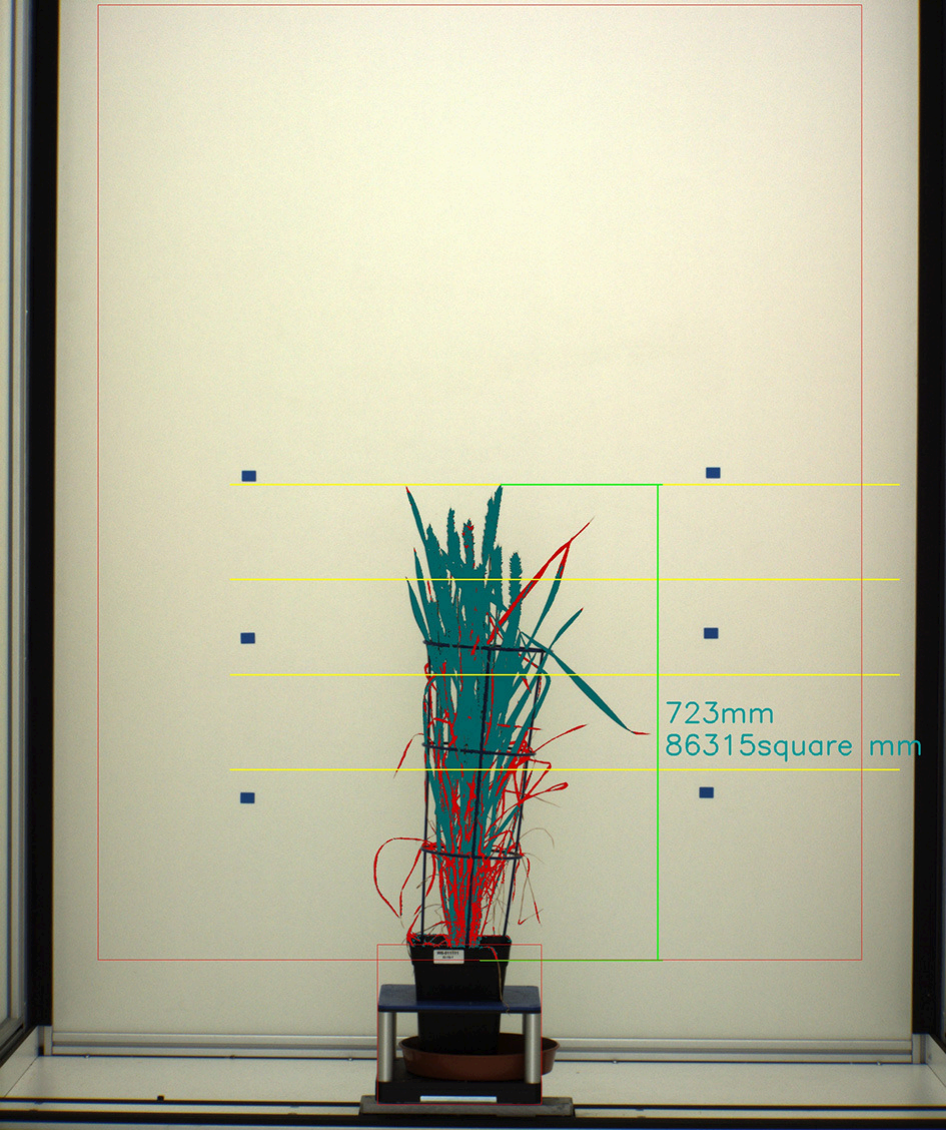
Segmentation of image showing a wheat plant. Plant Area is represented in dark green and red. Plant Senescence is represented by red. Height is represented by vertical green line.

Digital images were processed using C++ (software available at https://github.com/NPPC-UK/PAT64V3_W8). Briefly, image color classification was used to separate plant from background and a binary image was produced where “1” corresponded to plant and “0” to anything else. Then, image features accounting for plant height, plant area and plant yellow area were calculated from the analysis of “1” area. Height was measured from the surface of the soil (the front edge of the pot) to the uppermost part of the plant. Area was the total amount of segmented area. Yellow area was the total amount of regions of yellow color in the segmented area and was used as a proxy for Senescence (Figure 1).

### Statistical and quantitative trait locus analysis

Data analytics was performed using the R environment. QTL mapping was performed using the R package “HAPPY” for the analysis of multi-parental populations (Mott et al., 2000). SNP genotypes were used to infer ancestral haplotype mosaics for each MAGIC line and Single Marker analysis of variance was used to identify marker-trait associations. At each marker, lines were grouped according to their genotype and one-way ANOVA was used to test for significant differences between the groups. Marker-QTL association was indicated by F-statistics.

Significance for a given trait was tested by a permutation test (Camargo et al., 2008). First, the test statistic is calculated on the original data set. Next, phenotypes between lines are randomly shuffled 1,000 (B) times and test statistics are calculated on each shuffled data set. Finally, significance is estimated as the number of times (K) the test statistic value obtained in the original data set was smaller than those of the shuffled data sets, and dividing that value by the number of random shuffles, i.e., K/B. We used max(–logP) > 4 and *P* < 0.05 as a cut-off value in the multiple QTL analysis to test for association (Storey and Tibshirani, 2003).

### Data processing

Data extracted from digital images were subject to pre-processing. First, time points were discarded if the whole set of RILs and parents were not imaged. Second, Cook's distance (>4 μ) was used to identify and remove outliers.

### Genetic model for longitudinal traits

A trait is called time-dependent, or longitudinal, if measured over time. Time-dependent traits can be analyzed by; (1) using a repeated measurements framework; (2) treating phenotypes measured across time as different traits or; (3) fitting growth curves to the phenotypic values measured over time then analyzing the parameters describing growth's trajectory (Yang et al., 2006). This study fitted growth curves because they reduced the dimensionality of the data and treated phenotypes measured across time as different traits without dismissing the correlation between them.

Many of the complexities of plant growth are commonly represented using non-linear growth models. Growth rates calculated this way can capture age- and size-dependent growth (Paine et al., 2012). To identify the growth model that best fitted our data, we fitted logistic (Equation 1), 4-parameter logistic (Equation 2) and Gompertz (Equation 3) models to the average of area, height, senescence and water use. In addition, mixture distributions were fitted using finite mixture models to estimate the parameters that best described the modes (Equations 4–7). The best growth model was selected by comparing coefficient of determinations (Equation 8) from each model.

The equations for each growth model are as follows:

### Logistic model

(1) $$\begin{array}{l} f\left(t\right)=\;\frac{L}{\left(1+e^{-k\left(t-t_{0}\right)}\right)} \end{array}$$

where *L* is the upper asymptote, *k* is the growth rate and *t*_*0*_ is the value of *t* of the sigmoid curve's midpoint.

### 4-parameter logistic model

(2) $$\begin{array}{l} f\left(t\right)=D+\frac{\left(A-D\right)}{1+{\left(\frac{t}{C}\right)}^{B}} \end{array}$$

*A* and *D* are the bottom and top asymptotes (or the minimum and maximum values), *C* is the inflection point (or the point on the S shaped curve halfway between A and *D*), and *B* is the hill's slope, or the steepness of the curve. It could either be positive or negative.

### Gompertz model

(3) $$\begin{array}{l} f\left(t\right)=ae^{-be^{-ct}} \end{array}$$

where, *a* is an asymptote, ($\underset{t\to \;\infty }{\lim}ae^{-be^{-ct}}=ae^{0}=a$), *b, c* are positive numbers *b* sets the displacement along the time axis *t* and *c* sets the growth rate (y scaling).

### Finite mixture model

A finite mixture model (FMM) (Hathaway, 1985) is a probabilistic model for representing the presence of subpopulations (e.g., bi-modal distributions) within an overall population. In an FMM, the observed responses *y* are assumed to come from *m* distinct classes f_1_, f_2_,…, f*m* in proportions π_1_, π_2_,…, π_*g*_. In its simplest form, the density of a m-component mixture model as:

(4) $$\begin{array}{l} h\left(y|x,\psi \right)=\;\overset{m}{\underset{i\;=\;1}{\sum }}\pi_{m}f\left(y|x,\theta_{i}\right) \end{array}$$

where *y* is a dependent variable with conditional density *h, x* is a vector of independent variables, π_*i*_ is the prior probability of component *k*, θ_*i*_ is the component specific parameter vector for the density function *f*, and ψ = (π_i_, …, π_i_, θ′ _m_, …, θ′_m_ is the vector of all parameters (Hathaway, 1985).

The posterior probability that observation (*x*,*y*) belongs to class m is given by:

(5) $$\begin{array}{l} P\left(m|x,y,\;\psi \right)=\;\frac{\pi_{m}f\left(y|x,\;\theta_{m}\right)}{\sum_{k}\pi_{k}f\left(y|x,\;\theta_{k}\right)} \end{array}$$

The log-likelihood of a sample of N observations {(*x*_1_, *y*_1_), …, (*x*_*N*_, *y*_*N*_)} is given by:

(6) $$\text{logL}=\underset{n\;=\;1}{\sum^{N}}logh\left(y_{n}|x_{n,}\;\psi \right)=\underset{n\;=\;1}{\sum^{N}}log\left(\underset{i\;=\;1}{\sum^{m}}\pi_{i}f(y_{n}|x_{n,}\;\theta_{i}\right)$$

FMM uses the Expectation–maximization (EM) algorithm to refine starting values before maximum likelihood estimation via two steps:

**Estimate-step:** estimate the posterior class probabilities for each observation:
  $$\begin{array}{l} {\hat{p}}_{nk}=P\left(k|x_{n},\;y_{n},\;\hat{\psi }\right) \end{array}$$
Using Equation (2) and derive the prior class probabilities as
  $$\begin{array}{l} {\hat{\pi }}_{k}=\;\frac{1}{N}\overset{N}{\underset{n\;=\;1}{\sum }}{\hat{p}}_{nk} \end{array}$$
**Maximize-step:** maximize the log-likelihood for each component separately using the posterior probabilities as weights:
  (7) $$\begin{array}{l} \max\left(\theta_{k}\right)\overset{N}{\underset{n\;=\;1}{\sum }}{\hat{p}}_{nk}logf\left(y_{n}|x_{n,\;}\theta_{k}\right)\; \end{array}$$

The E- and M-steps are repeated until either the likelihood improvement falls under a given threshold or a number of searches is reached.

Coefficient of determination (*R*^*2*^) is used for model selection:

(8) $$\begin{array}{l} 1-\frac{\sum_{i}{\left(y_{i}-{ŷ}_{i}\right)}^{2}}{\sum_{i}{\left(y_{i}-{ȳ}_{i}\right)}^{2}} \end{array}$$

### Heritability

Growth curve parameters were estimated for each trait and for each plant, and parameters' heritability (*H*^2^) (Equation 9) and genetic (*g*) and environmental (*e*) covariances (Equation 10) and correlations (Equation 11) between parameters were estimated.

(9) $$\begin{array}{l} H^{2}\left(x\right)=\;\frac{s_{g}^{2}\left(x\right)}{s_{g}^{2}\left(x\right)+s_{e}^{2}\;\left(x\right)} \end{array}$$

Where *H*^2^ is the heritability for a given trait, *x*, and $s_{g}^{2}$(*x*) and $s_{e}^{2}\left(x\right)$ are the genetic and environment variances for that trait.

(10) $$\begin{array}{l} S\left(x,y\right)=\;\frac{s_{i}^{2}\left(x+y\right)-s_{i}^{2}\left(x\right)-s_{i}^{2}\left(y\right)}{2\;} \end{array}$$

Where *S* is the covariance between two traits (*x,y*), *s*^2^ is the variance and *i* = *g* or *e*, genetic or environment

(11) $$\begin{array}{l} r\left(x,y\right)=\;\frac{S\left(x+y\right)}{\sqrt[{2}]{s^{2}\left(x\right)+\;s^{2}\left(y\right)}\;} \end{array}$$

where *r* is the correlation between two traits (*x,y*).

## Results

### Trait analysis

**Figure 2:**
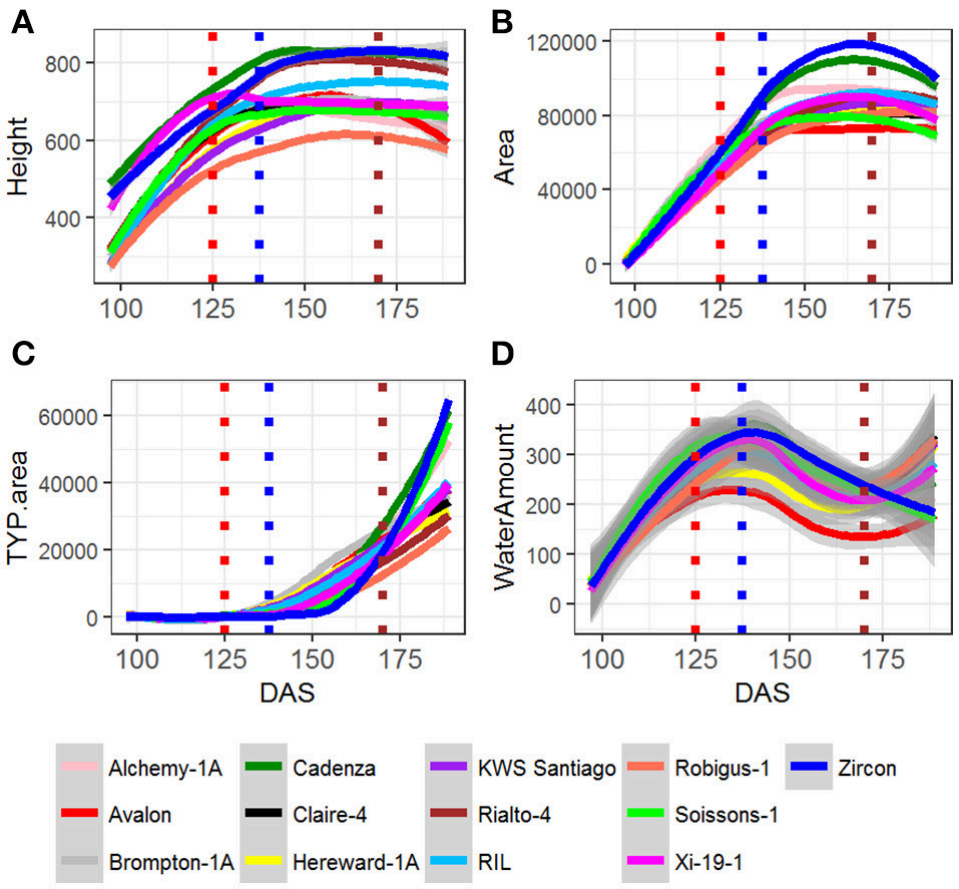
Fitted curves for MAGIC parents and all RILS, for **(A)** Height, **(B)** Area, **(C)** TYP. Area Senescence, and **(D)** Water use. RIL is the average of all RILs per time point. For all the plants, red dotted line indicates average DAS for GS39, blue dotted line indicates average DAS for GS55 and brown dotted line indicates average DAS for FLS.

Analysis of trait across time indicated that in general all the MAGIC lines and their parents maintained steady growth and water use up until 138 DAS (Figures 2A–C, blue dotted line) which coincided with the timing at which most of the plants reached GS55 (~136 DAS) and started showing signs to senescence, as indicated in Camargo et al. (2016). Figure S1 shows the average pattern of leaf senescence plotted against the average of water consumption for all plants. The distribution of the curves suggests that the onset of senescence can be predicted from a plant's water consumption.

In addition, the time point at which the Water Use curve starts to rise for the second time, 170 DAS (Figure 2D, brown dotted line), also coincides with the time at which most plants initiate senescence at the Flag leaf (170 DAS) (Figure 2D).

### Growth curve analysis

**Figure 3:**
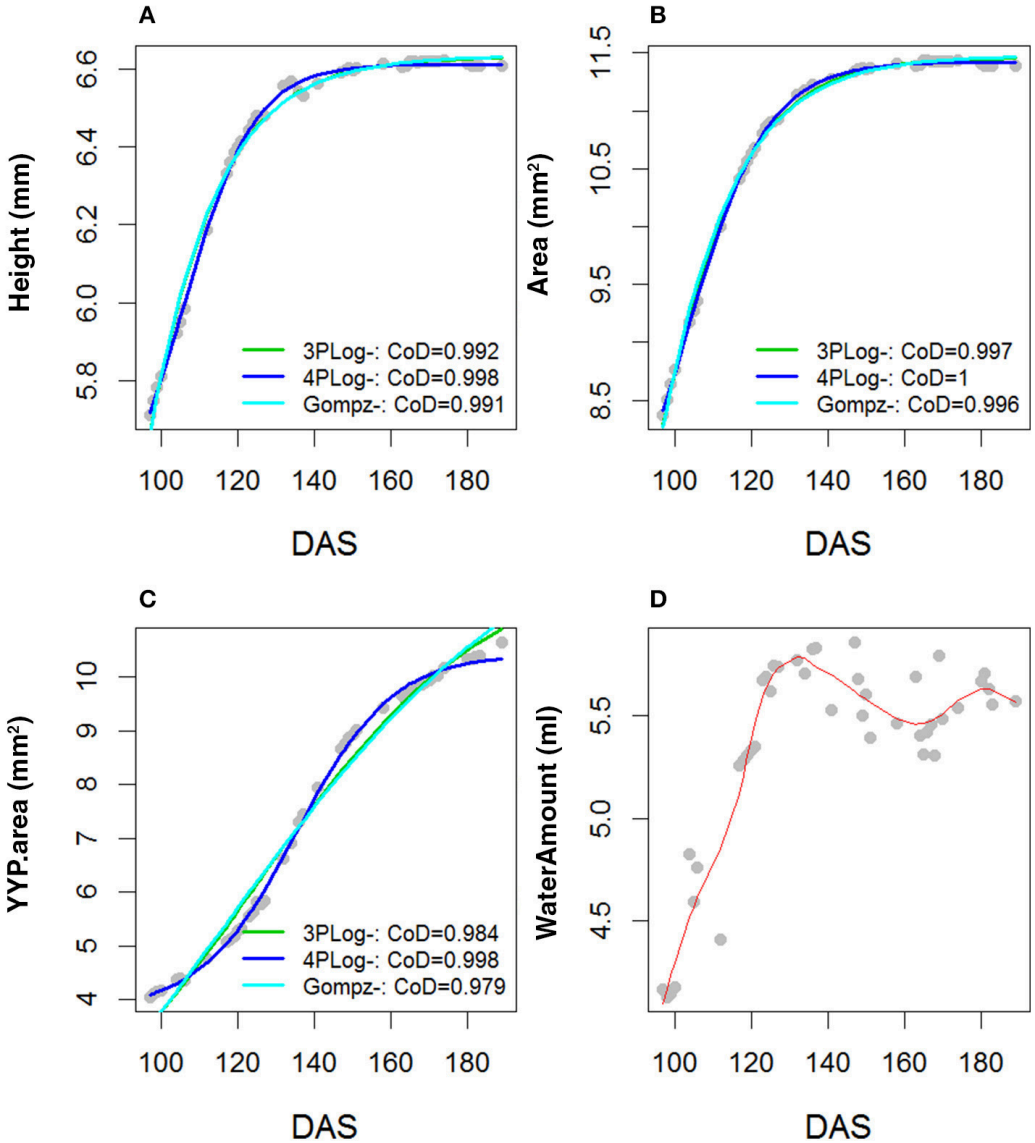
Comparison of *R*^2^ across three parameter logistic, four parameters logistic and Gompertz. Growth models were fitted to the population mean and for the traits **(A)** Height, **(B)** Area, **(C)** TYP. Area (Senescence) and **(D)** Water Amount. The bi-modal shape of the water amount curve was fitted with a Gaussian type growth model.

Area, Height and Senescence (TYP) showed a logistic growth (Figures S3–S9). Figures 2A–C plots average values of these three traits over time for founder lines, controls and the mean of the RILs. For the population mean, comparison of error sum of squares from the fitted values of several growth models indicated that these traits were best described by a 4-parameter logistic growth curve (Equation 2; Figures 3A–C).

Growth parameters (Equation 2) were estimated for each trait and for each plant. Using data from the RILs, the heritability of these parameters and their genetic and environmental co-variances and correlations were estimated. Results are shown in Tables S1–S6.

In the analysis of Area and Height growth curve parameters, *B* (the curve's slope) has the highest heritability (>0.8). The remaining parameters have heritability below 0.5 indicating that the rate at which a plant grows is highly heritable. However, *B* did not show strong genetic or environmental correlations with the other parameters. *A* and *C* (the bottom asymptote and inflection point, respectively) were highly correlated both genetically and environmentally (*r* > 0.9) and *A* and *D* were negatively correlated (<−0.6) at the genetic level. Senescence growth curve parameters did not show high heritability for any parameter. However, the genetic and environment correlations between *A* and *D* were negative (<−0.6) and there was a large positive environment correlation (0.9) between *B* and *D*.

In comparison to Area, Height and Senescence's curves, the Water Use curve was bi-modal (Figures 2A–C). The peak of the first mode was around 130 DAS and for the second around 180 DAS. Given the bi-modality, a mixture model (Equation 4) was fitted to the data to estimate the parameters describing the two modes; their amplitudes, λ, means, μ, and standard deviations, σ (Figure 3). The separation (Zhang et al., 2003) between the two modes was calculated as:

(12) $$\begin{array}{l} S=\;\frac{\mu_{2}-\mu_{1}}{\left(2\sigma_{1}+2\sigma_{2}\right)} \end{array}$$

Growth parameters μ_1_, λ_1_, and σ_1_, corresponding to the first mode, μ_2_, and σ_2_, corresponding to the second mode, were estimated for each trait and for each plant. Parameters for the RILs were then used to estimate heritabilities, co-variances and correlations. Results shown in Tables S7, S8 indicate that none of the growth parameters were highly heritable (*H*^2^ < 0.2). With such low heritabilities, genetic correlations are very poorly estimated and correlations between parameters are largely environmental. The pattern of these correlations indicates that early peak Water Use (smaller μ_1_) is associated with more water being used in the early period (high λ_1_, and σ_1_) and that this delays the second water uptake phase (high μ_2_). Xie et al. (2015) identified a similar Water Use peak after anthesis, and identified positive relationships between maximum Senescence rate and the time for maximum grain dimensions. We also compared thermal time against Water Use after anthesis and found a similar peak after that stage (Figure S2) which confirms the two peaks observed in the Water Use curve.

### Quantitative trait loci

The lines were genotyped using the Illumina Infinium iSelect 80,000 SNP wheat array (“80 K array,” http://www.illumina.com/), described in Yang et al. (2006). Prior to QTL mapping, a test for population structure was performed as indicated in Camargo et al. (2016).

### Analysing individual time points

**Figure 4:**
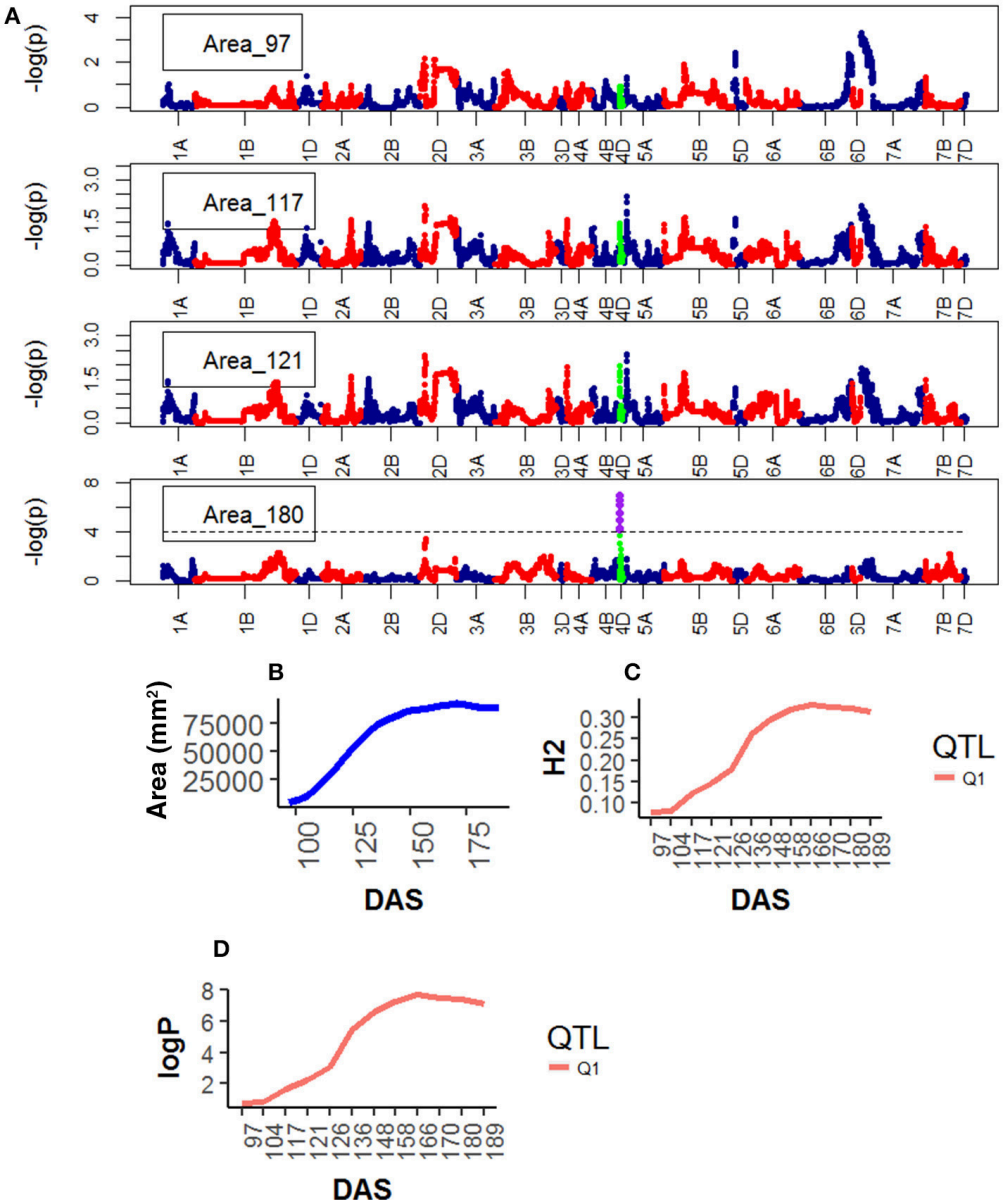
Longitudinal analysis of the trait Area. **(A)** Manhattan plots highlighting chr4D at four DAS. Purple dots indicate QTLs with *P*-value < 0.05 and max(–logP) > 4; **(B)** Population Area mean **(C)** heritability (H^2^) for peak QLTs (Table 2), **(D)** –logP.

**Figure 12:**
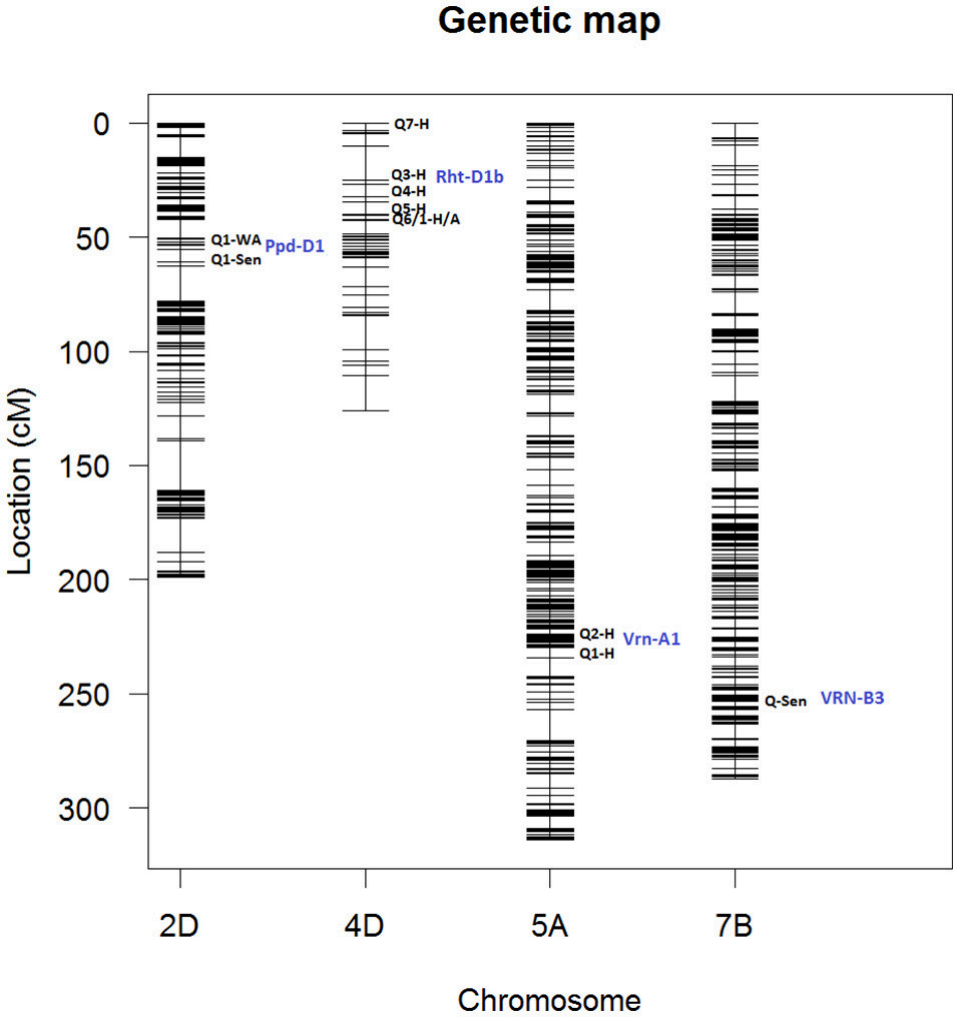
Genetic map showing QTL locations for chromosomes 2D, corresponding to markers: (Q1) GENE_0137_147 and (Q1) Kukri_c27309_590; 4D corresponding to markers: (Q7) Tdurum_contig420, (Q3) Kukri_rep_c68594_530, (Q4) RAC875_c1673_193, (Q5) RAC875_c6922_291, (Q6/Q1) RAC875_rep_c105718_585; 5A, markers: (Q2) IAAV1650 and (Q1) BS00011360_51 and 7B, markers: BS00087197_51. Blue labels indicate the names associated to each chromosome location. Q# indicate QTL number in Table 2. H, Height; TYArea, Senescence; A, Area; WA, Water Amount. More information about these QTLs is given in Table 2.

Analysis of individual phenotypes at each time point identified loci associated with all traits but not with all the time points. For Area, seven significant QTLs were found on chr4D (Table S9), peak QTL corresponded to the marker RAC875_rep_c105718_585, between 11.0 and 23.0 Mb (90% confidence region) (Table 2, Figure 12). This QTL became highly significant at around 136 DAS and reached its maximum statistical significance at 166DAS (Figure 4).

**Table 2:** Peak QTLs from the mapping of individual time points and growth curve parameter for Area, Height, Senescence and water use (WA).

**Trait**	**DAS**	**peak.SNP**	**SN**	**chr**	**cM**	**Alchemy**	**Brompton**	**Claire**	**Hereward**	**Rialto**	**Robigus**	**Soissons**	**Xi19**	**h2**	**logP**	***P*-value**	**Lower**	**Upper**
Area	136	RAC875_rep_c105718_585	Q1	chr4D	40.11	69244.19	70726.64	68961.25	71277.8	64324.87	88639.24	82420.8	68635.23	0.26	5.03	0	1100001	2100001
Area	148	RAC875_rep_c105718_585	Q1	chr4D	40.11	79011.69	80634.7	79132.84	79329.72	72228.77	104657.47	95155	78610.13	0.30	6.13	0	1100001	2200001
Area	158	RAC875_rep_c105718_585	Q1	chr4D	40.11	83601.38	84625.25	83505.17	82474.3	74737.2	110709.22	99681.57	81966.03	0.32	6.84	0	1100001	2300001
Area	166	RAC875_rep_c105718_585	Q1	chr4D	40.11	85421.27	86303.78	84829.58	85063.95	76910.64	115352.03	101544.8	84263.48	0.33	7.24	0	1100001	2300001
Area	170	RAC875_rep_c105718_585	Q1	chr4D	40.11	84679.14	86124.21	85332.53	84096.08	76355.65	114026.02	100925.26	84268.74	0.32	7.01	0	1100001	2200001
Area	180	RAC875_rep_c105718_585	Q1	chr4D	40.11	84520.25	83636.76	84650.83	83425.9	75116.47	113026.45	97528.08	81995.76	0.32	6.98	0	1100001	2300001
Area	189	RAC875_rep_c105718_585	Q1	chr4D	40.11	82732.5	82136.26	83503.19	81775.89	72993.25	111018.8	94719.31	81649.07	0.32	6.69	0	1100001	2300001
Height	104	IAAV1650	Q2	chr5A	227.15	359.21	351.54	372.03	365.19	366.47	370.33	365.25	483.14	0.38	8.5	0	62600001	78600001
Height	117	Kukri_rep_c68594_530	Q3	chr4D	24.93	549.21	552.46	547.53	529.87	546.62	605.29	602.12	538.92	0.32	6.9	0	1000001	2100001
Height	117	Tdurum_contig42083_1539	Q7	chr4D	3.09	560.86	577.26	562.7	546.19	547.5	582.53	573.2	580.93	0.28	5.52	0	100001	200001
Height	121	RAC875_c1673_193	Q4	chr4D	32.24	587.3	595.07	586.07	585.94	586.81	656.97	665.65	574.99	0.25	4.71	0.01	1500001	2100001
Height	121	RAC875_c6922_291	Q5	chr4D	26.97	599.51	601.28	600.33	593.75	600.11	649.53	641.04	580.64	0.30	6.2	0	1000001	1300001
Height	121	Tdurum_contig42083_1539	Q7	chr4D	3.09	610.88	620.54	608.56	609	594.23	629.3	621.7	627.37	0.23	4.09	0.04	100001	200001
Height	126	RAC875_c6922_291	Q5	chr4D	26.97	635.91	639.62	634.65	629.29	636.49	688.47	678.17	617	0.34	7.59	0	1000001	2100001
Height	136	RAC875_c1673_193	Q4	chr4D	32.24	665.19	672.13	664.86	668.97	670.64	748.67	752.33	655.72	0.32	6.84	0	1000001	2200001
Height	148	RAC875_rep_c105718_585	Q6	chr4D	40.11	690.27	741	689.67	695.06	699.69	826.91	798.72	687.68	0.41	10.12	0	1000001	2800001
Height	158	RAC875_c1673_193	Q6	chr4D	32.24	709.59	718.92	705.97	713.13	703.46	837.5	820.89	691.51	0.42	10.47	0	1000001	2800001
Height	166	RAC875_c1673_193	Q4	chr4D	32.24	706.59	716.82	715.03	709.11	708.93	855.32	839.75	695.3	0.40	9.62	0	1000001	2500001
Height	170	RAC875_c1673_193	Q4	chr4D	32.24	711.2	723.01	711.97	709.62	708.92	860.57	835.8	693.25	0.40	9.75	0	1000001	2500001
Height	180	RAC875_c1673_193	Q4	chr4D	32.24	705.56	713.01	710.01	708.75	700.14	856.26	831.98	691.63	0.40	9.77	0	1000001	2500001
Height	189	RAC875_c1673_193	Q4	chr4D	32.24	703.12	712.39	697.06	704.36	697.64	850	831.53	681.1	0.41	10.02	0	1000001	2400001
Height	97	BS00011360_51	Q1	chr5A	229.67	290.19	287.85	302.31	289.38	299.26	296.85	299.37	374.83	0.31	6.16	0	62700001	78500001
TYArea	166	Kukri_c27309_590	Q1	chr2D	55.4	20203.98	18583.62	20025.34	20084.7	18496.39	18941.17	13170.77	19521.4	0.25	4.2	0.03	13800001	13900001
TYArea	180	RAC875_c1673_193	Q2	chr4D	32.24	27708.2	27568.07	27379.38	27410.43	26809.1	38035.26	38044.2	25882.88	0.26	4.9	0.01	1300001	2100001
TYArea	189	RAC875_c1673_193	Q2	chr4D	32.24	36642.01	36791.6	36739.89	37302.61	35809.98	52920.44	51199.04	35243	0.33	7.04	0	1100001	2100001
WA	158	GENE_0137_147	Q1	chr2D	53.36	242.79	233.5	237.44	241.51	240.05	244.43	203.84	241.79	0.25	4.19	0.02	12500001	13700001
At		BS00087197_51		chr7B	253.51	3.82	3.6	3.58	4.05	3.59	2.78	3.05	3.82	0.25	4.25	0.05	74600001	74700001
B		RAC875_rep_c105718_585		chr4D	40.11	11.29	11.3	11.3	11.31	11.2	11.63	11.48	11.31	0.31	6.58	0	1100001	2200001
Bh		RAC875_c1673_193		chr4D	32.24	6.53	6.54	6.52	6.55	6.54	6.75	6.68	6.52	0.38	9.09	0	1100001	2500001

*cM is the marker position. h^2^ is heritability. logP is –log10 at the QTL peak; chr is the chromosome. P-value is the genome wise P-value for the QTL based on permutations. Alchemy, Brompton, Claire, Hereward, Rialto, Robigus, Soissons and Xi-19 indicate the estimated founder QTL effects as produced by HAPPY. Last two columns show 90% confidence interval (CI) for the QTL based on permutations. These values are island intervals, they are the segments exceeding the genome-wide significance level*.

**Figure 5:**
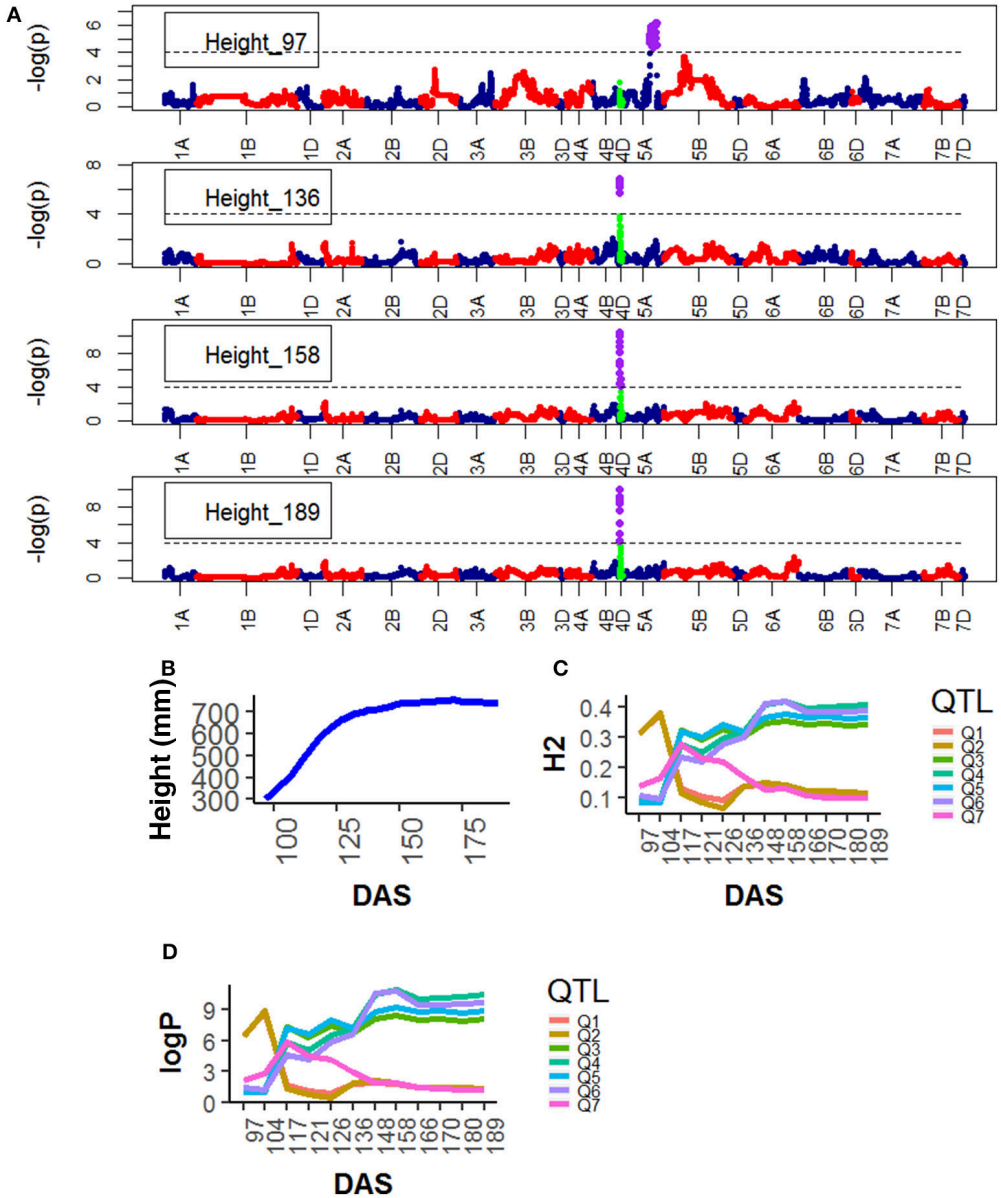
Longitudinal analysis of the trait Height **(A)** Manhattan plots highlighting chr4D at four DAS. Purple dots indicate QTLs with *P*-value < 0.05 and max(–logP) > 4; **(B)** Population Area mean **(C)** heritability (H^2^) showing peak markers (Table 2) per chromosome [*P* < 0.05, max(–logP)], **(D)** LOD scores [max(–logP)] showing peak markers per chromosome [*P* < 0.05, max max(–logP)].

For Height, the following QTLs were identified across different chromosomes: 13 were found on chr4D (Table S9), peak QTLs corresponded to the markers Kukri_rep_c68594_530 (10.0–21.0 Mb), RAC875_c6922_291 (10.0–13.0 Mb), RAC875_c1673_193 (15.0–21.0 Mb), RAC875_rep_c105718_585 (11.0-23.0 Mb) and Tdurum_contig42083_1539 (10.0–20.0 Mb). Two were found on chr5A corresponding to wsnp_Ra_c44756_51084202 and wsnp_Ex_c113235_94249366. Two were found on chr5A corresponding to IAAV1650 (62.6–78.6 Mb) and BS00011360_51 (62.7–78.5 Mb). The statistical significance of the QTLs on chr5A was high (>6.0 logP) at early plant development (~ 97 DAS) and decreased as the plant matured. The QTLs on chr5B showed a similar pattern but their statistical significance was low. In contrast, the statistical significance of the QTLs on chr4D was low (< 2.0 logP) during early plant development (~97 DAS) but became high around 136 DAS and reached their maximum significance level at 158 DAS which coincided with the time the average plant reached its maximum growth (Figure 5).

**Figure 6:**
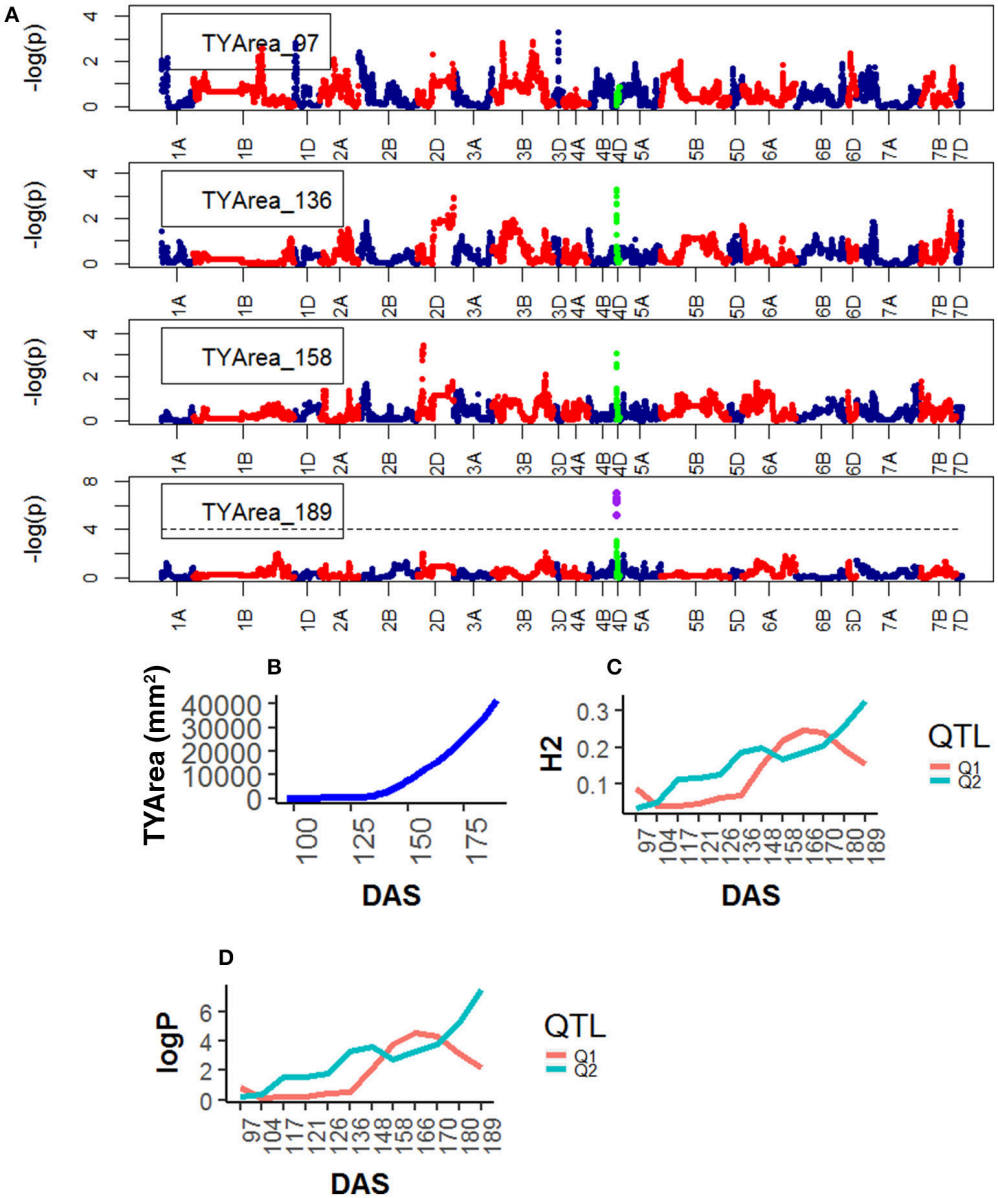
Time resolved analyses for the trait Senescence (TYP) **(A)** Manhattan plots highlighting chr4D at four DAS. Purple dots indicate QTLs with *P*-value < 0.05 and max(–logP) > 4; **(B)** Population Area mean **(C)** heritability (H^2^) showing peak markers (Table 2) per chromosome [*P* < 0.05, max(–logP)], **(D)** LOD scores [max(–logP)] showing peak markers per chromosome [*P* < 0.05, max(–logP)].

For Senescence, one QTL was found on chr4D, corresponding to the marker RAC875_c1673_193, and one on chr2D corresponding to Kukri_c27309_590.The statistical significance of the QTLs on chr4D was low (<2.0 logP) at early plant development (~97 DAS) but became high at around 148 DAS and reached their peak at 189 DAS, at the end of the plant's life (Figure 6).

**Figure 7:**
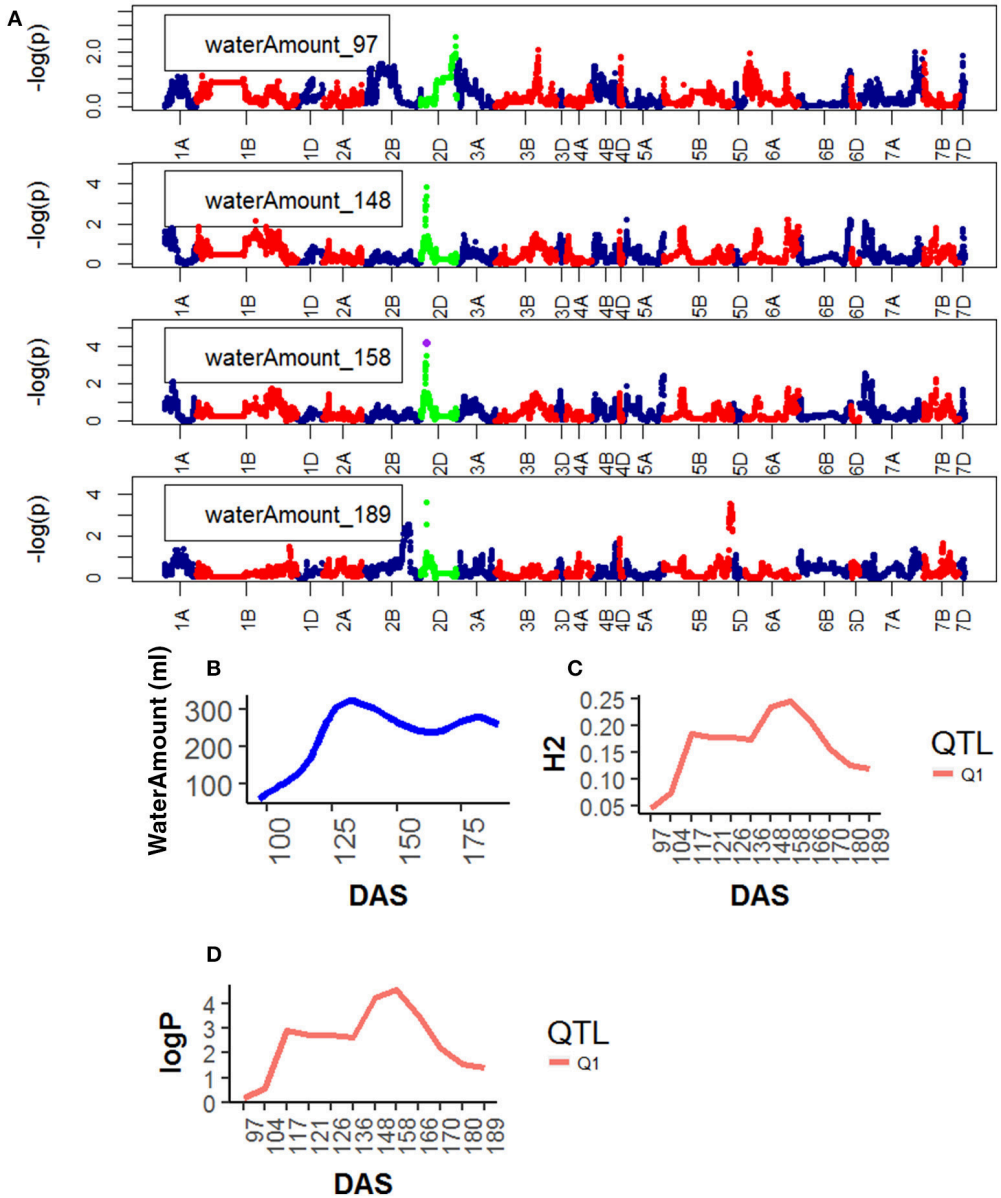
Time resolved analyses for the trait Water use (Water amount) **(A)** Manhattan plots highlighting chr4D at four DAS. Purple dots indicate QTLs with *P*-value < 0.05 and max(–logP) > 4; **(B)** Population Area mean **(C)** heritability (H^2^) showing peak markers (Table 2) per chromosome [*P* < 0.05, max(–logP)], **(D)** LOD scores (logP) showing peak markers per chromosome [*P* < 0.05, max(–logP)].

For Water Use, one QTL was found on chr2D, corresponding to the marker GENE_0137_147 (12.5–13.7 Mb). Significance level of the QTL on chr2D was high at around 136 DAS and reached its maximum at 166DAS (Figure 7), after that significance levels became lower.

QTL associated with RAC875_rep_c105718_585 on chr4D were common for Area and Height and with RAC875_c1673_193 on chr4D for Height and Senescence. Both QTL became highly significant at around 136 DAS.

Figure 12 shows locations of peak QTLs located on chromosomes 2D, 4A, 5A, and 7B. Most peak QTLs are clustered within short distance except for Tdurum_contig42083_1539 on chr 4A which is isolated for the main cluster.

### Analysing of growth curve parameter

**Figure 8:**
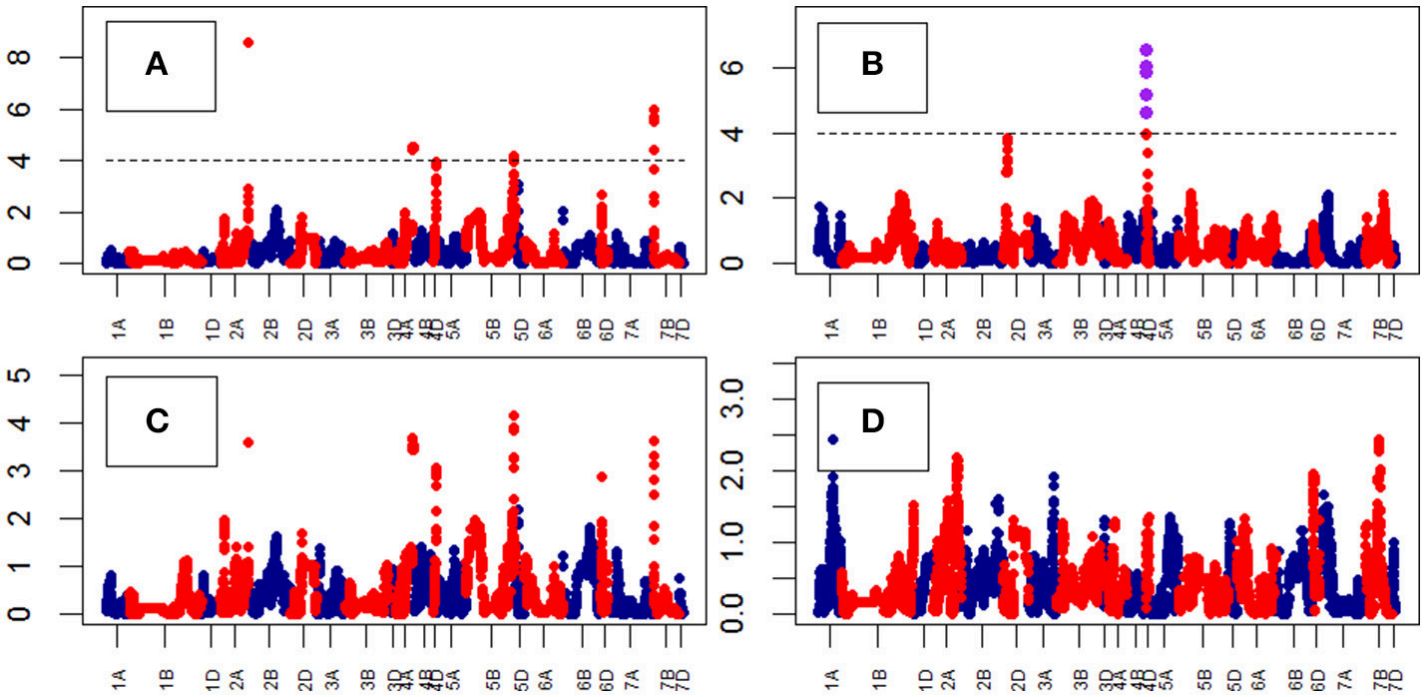
Manhattan plots from the genetic mapping of the curve's growth parameters corresponding to the trait Area. Purple dots indicate QTLs with *P*-value < 0.05 and max(–logP) > 4. **(A–D)** are the parameters derived from the 4-Parameter Logistic Model, and are provided in Table 1.

**Figure 9:**
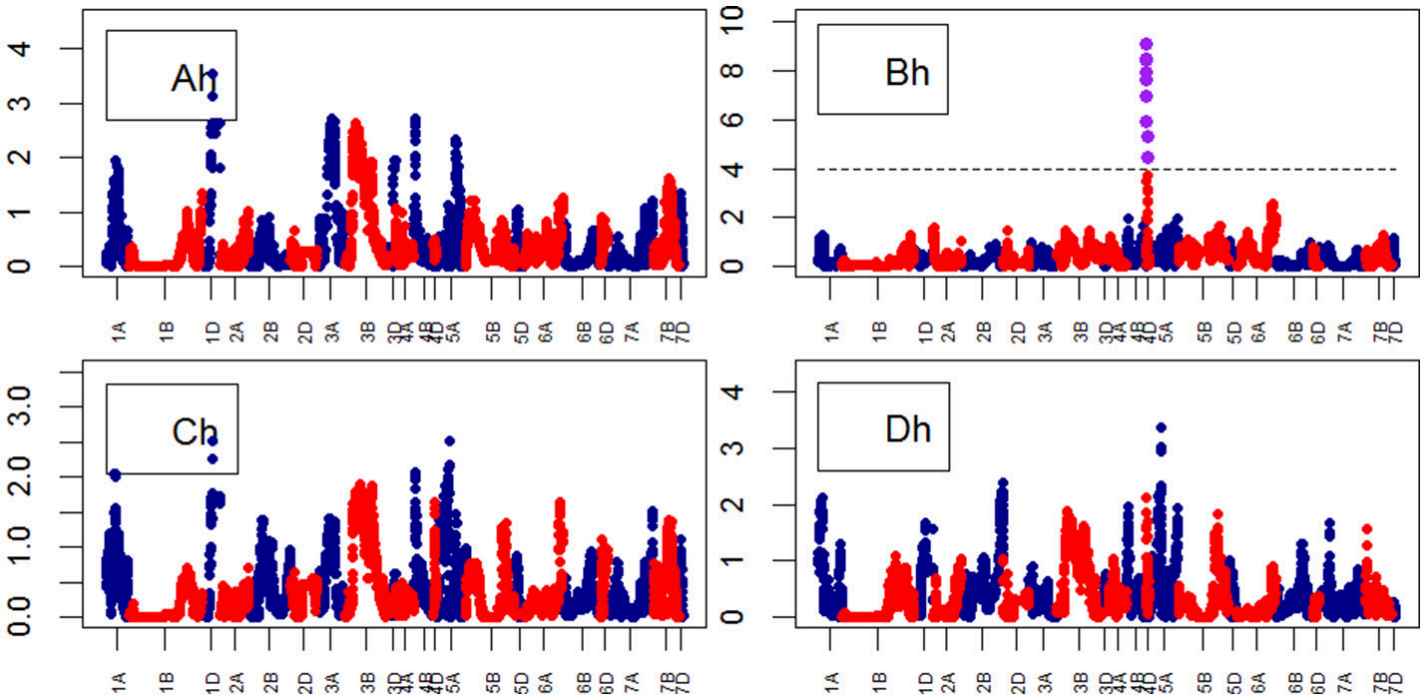
Manhattan plots from the genetic mapping of the curve's growth parameters corresponding to the trait Height. Purple dots indicate QTLs with *P*-value < 0.05 and max(–logP) > 4. **(Ah–Dh)** are the parameters (see Table 1) derived from the 4-Parameter Logistic Model for Height, and are provided in Table 1.

Growth curve parameters fitted to Area across time identified nine QTLs associated with the parameter “*B*” on chr4D (Table S9). The peak QTL was BS00022276_51 (Figure 8). The analysis of growth parameters for Height also identified 13 QTLs on chr4D. The peak QTL was RAC875_c1673_193 (Figure 9) which was also linked to Area and Height.

**Figure 10:**
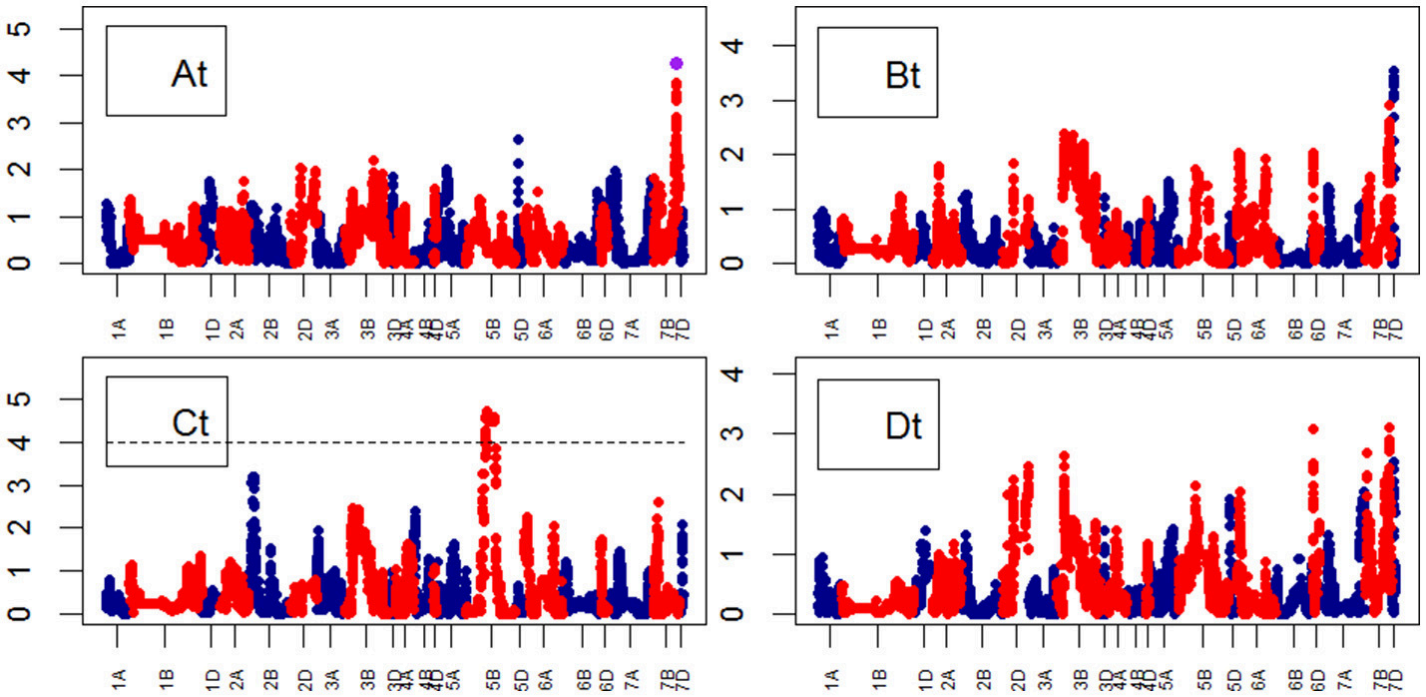
Manhattan plots from the genetic mapping of the curve's growth parameters corresponding to the Senescence (TYP) Area. Purple dots indicate QTLs with *P*-value < 0.05 and max(–logP) > 4. **(At–Dt)** are the parameters (see Table 1) derived from the 4-Parameter Logistic Model for TYP (Senescence), and are provided in Table 1.

**Figure 11:**
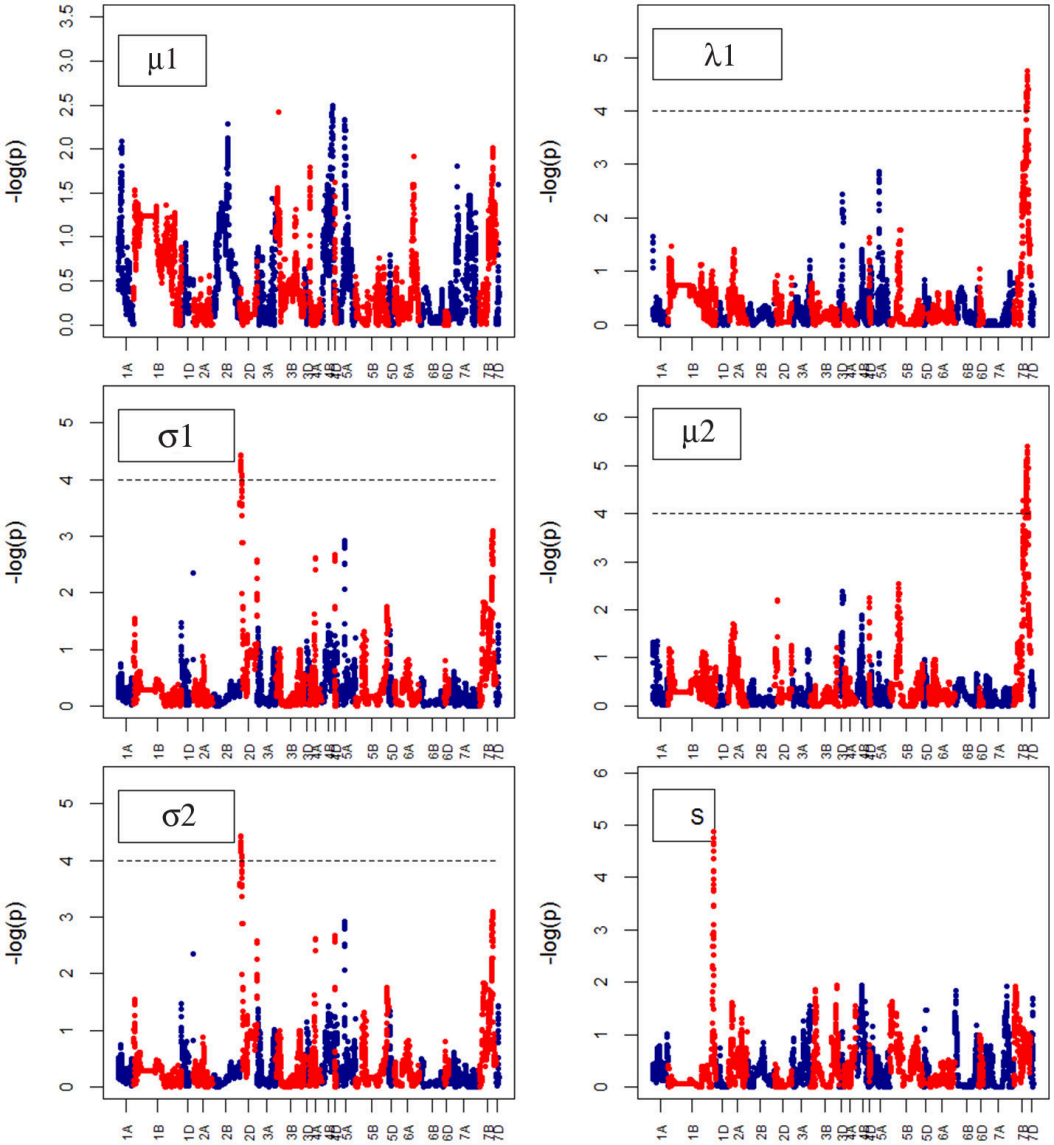
Manhattan plots from the genetic mapping of the curve's growth parameters corresponding to Water Use. μ1, λ1, σ1, σ2 and S are the parameters (see Table 1) derived from the (Polynominal logistic Model or Finite Mixture Model), and are provided in Table 1.

For Senescence growth parameters, one QTL on chr7B was associated to the “*A*” parameter at marker BS00087197_51 (74.6–74.7) (Figure 10). For Water Use growth parameters, we did not identify QTLs (Figure 11).

## Discussion

This work aimed to understand the genetic elements influencing the temporal and spatial changes in key traits such as plant height and area, the timing of senescence and the dynamics of water use. Since plant traits were measured across time, we used two different approaches to analyse growth trajectories: first, growth curves were fitted to the phenotypic values of each trait measured over time. Second, phenotypic measurements were treated as different traits and analyzed individually.

For the analysis of growth curves, we initially fitted second, third and fourth degree polynomials of the form $RGR\;=\;{\text{b}}_{0}\;+\;{\text{b}}_{1}\text{t }+\;{\text{b}}_{2}{\text{t}}^{2}\;+\;\ldots \;+\;{\text{b}}_{n}t^{n}$ to the longitudinal data (Poorter, 1989), and performed QTL mapping over the polynomials' coefficients. Polynomials curves are easy to fit because they use standard linear modeling procedures and they can also approximate non-linear functions relatively well. However, results of QTL mapping did not identify any marker association from any of the polynomials fitted to the longitudinal data.

Higher order polynomials are difficult to interpret and are unlikely to have an obvious biological meaning: they describe the form of the curve rather than offering any natural explanation. In contrast, the logistic have been used successfully to model growth, and the parameters better describe the biology of growth (Paine et al., 2012). That QTL are found for the growth curve parameters but not for any of (or the higher order) polynomial regression coefficients is in agreement with this interpretation.

After evaluating curve fitting by using polynomials, we fitted non-linear plant growth models to the longitudinal data and through the comparison of *R*^2^ identified the 4-parameter logistic model as the model that best fitted the phenotypic traits describing the growth trajectories of area, height and senescence. As explained later in this section, QTL mapping of curve parameters identified significant marker associations.

Comparison between growth curve parameters, identified curve steepness (“*B*”*)* as the most heritable (>0.8) for area and height but not for senescence. However, “*B*” was not correlated to any other parameter in contrast to “*A*” and “*C*,” which were highly correlated both genetically and environmentally (*r* > 0.9), indicating that phenotypic outcome was highly dependent on genotype.

Since the Water Use curve showed a different growth trajectory (Figure 3D), having two modes, a multi-modal modeling approach was used to model the heterogeneity of the Water Use curve. When affecting fitness related traits such as survival or reproduction, individual heterogeneity plays a key role in population dynamics and life history evolution. However, it is only recently that properly accounting for individual heterogeneity when studying population dynamics has been made possible through the use of high-throughput technologies and the development of appropriate statistical models (Ford et al., 2015; Gimenez et al., 2017). In this study, we used a similar approach to Bresson et al. (2015) to model the bi-modality of the curve trajectory describing Water Use. Although, the analysis of growth parameters did not identify highly heritable parameters or high correlations between them, we realized the curve trajectory followed a similar pattern of observed by Xie et al. (2015) when they were looking into water accumulation and grain weight in wheat. The low heritability of Water Use growth parameters could also be explained by the sensitivity of water use to environmental variables such as changes in VPD.

Genetic mapping of individual phenotypic values measures across time not only highlighted key markers associated with area, height, senescence and water use but also indicated when marker effects became especially large or when their signal decayed. Most importantly, when comparing the plots on Figure 2 and those on Figures 4–7B,C, one can assess how trait development correlates with relative QTL strength.

Genetic mapping analysis identified peak QTLs for the traits Senescence (Q1) and Water Use (Q1) on chr2D at the location of *Ppd-D1*, the photoperiod sensitivity (Bentley et al., 2013) locus. The effect was highly significant at around 148 DAS and became less significant after that. In addition to the mapping of phenological traits, we also looked for markers associated with morphological traits. For example, strong markers on chr4D such as RAC875_rep_c105718_585 and RAC875_rep_c105718_585 were linked with plant Area and plant Height. In both cases, the QTL interval includes the *Rht-D1* (*Rht2*) locus. The *Rht-D1b* allele at this locus has been associated with a reduction in plant height and several other morphological traits and is segregating in the MAGIC population (the dwarfing allele at this locus is present in all parent lines except Robigus and Soissons, which carry the dwarfing allele *Rht-B1b*). Table 2 confirms that the estimated founder QTL effect contribution of Robigus and Soissons over height and for the RAC875_rep_c105718_585 marker is higher than for the other parents.

We also identified two strong effects (–logP > 8.00) on chr5A associated with plant Height at markers IAAV1650 and BS00011360_51. We believe these markers are closely linked to the vernalization gene *Vrn-A1* which plays an important role in the vernalization process in diploid Einkorn wheat (*Triticum monococcum*) and polyploid common wheat (*Triticum aestivum*; Kiss et al., 2014). Finally, a peak marker was found on chr7B which was associated to Senescence. Table 2 shows that the estimated founder QTL effect contribution Xi19 over Height and for the IAAV1650 and BS00011360_51 markers is higher than for the other parents. BS00011360_51 is likely to be the VRN-B3 gene which has been associated with increased yield in EU 2011 and GBR 2010 wheat trials (Bentley et al., 2014).

To look further into the effect of key markers over trait development, we fitted a regression model of the trait against the marker, for each time point. Results are summarized in Figures S7–S11. Briefly, RAC875_rep_c105718_585 (likely to be the *Rht-B1b* gene) marker showed a allelic effect on area, height, senescence and negative on Water Use, indicating a positive correlation with the trait. The Kukri_c27309_590 marker (likely to be related to the *Ppd-D1* gene) showed a positive effect on area, height, and water use, indicating a positive correlation with the trait. However, for Senescence, the marker effect was positive up until 136 DAS but its significance level was lower after that time point which coincided with the average plant initiating Senescence. The marker IAAV1650 (closely linked to the vernalization gene *Vrn-A1*) showed completely different patterns for each trait: for Area, the effect was positive; for height, the effect was negative; for senescence the effect became positive after the average plant started senescence; and for water use the effect became negative at around 158 DAS. The BS00087197_51 (on chr7B) showed a positive effect on area and water use and negative on height. However, for senescence, the marker effect was negative up until 136 DAS but its significance level was lower after that time point, this pattern was the opposite of that showed by the Kukri_c27309_590 marker. Regardless of the marker, this analysis together with QTL mapping seems to suggest that fundamental changes take place around 136 DAS.

Genetic mapping of growth curves parameters fitted to the same traits confirmed the identification of major QTLs such as those on chr2D, chr4D, and on chr7B. This result demonstrate the power of analyzing growth curves that carry all the information about the plant's development as opposed to the analysis of single time points. However, it also showed that the analysis of single time points helps to describe how traits and their marker effects evolve over time. Figure S11 shows a comparison of phenotypes vs. haplotypes of a number of RILS who carried different allelic variation for the markers m1 = RAC875_rep_c105718_585, m2 = BS00011360_51, m3 = IAAV1650 and m4 = Kukri_c27309_590. Although these four large QTL have been detected in this experiment and they are known important controllers of plant growth and architecture in wheat (*Vrn-A1, Rht-D1*, and *Ppd-D1*), there are no striking differences between these 16 plants representing all possible combination. This demonstrates the merit of accurate measurement taken here to quantify the parameters responsible for plant form and development.

This study used a new data driven approach to analyse the longitudinal data capturing the process of formation of dynamic traits such as senescence or water use. We combined the mapping power and precision of a MAGIC wheat population with robust computational methods to track the spatio-temporal dynamics of traits associated with wheat performance. We demonstrated that the combination of these two methodologies can be used as a powerful strategy for fine-tuning wheat's response to the environment.

## Author contributions

AC and IM conceived analysis. AB and JD developed and obtained funding for the project. JH processed digital images. AC analyzed data. RM, IM, and AC assisted with the analysis. KA, FC, and KW provided technical support in the collection of phenotypic data. All authors provided comments and corrected the manuscript.

### Conflict of interest statement

The authors declare that the research was conducted in the absence of any commercial or financial relationships that could be construed as a potential conflict of interest.
