# Supplementary material for: Functional Mapping of Quantitative Trait Loci (QTLs) Associated With Plant Performance in a Wheat MAGIC Mapping Population
Source: Front Plant Sci. 2018 Jul 9;9:887. doi: 10.3389/fpls.2018.00887 (PMC6047115; doi:10.3389/fpls.2018.00887)
Supplement: Supplementary file 1 [file Image_1.PDF]

Functional mapping of quantitative trait loci (QTLs) associated with plant performance in a wheat MAGIC mapping population

Supplementary material

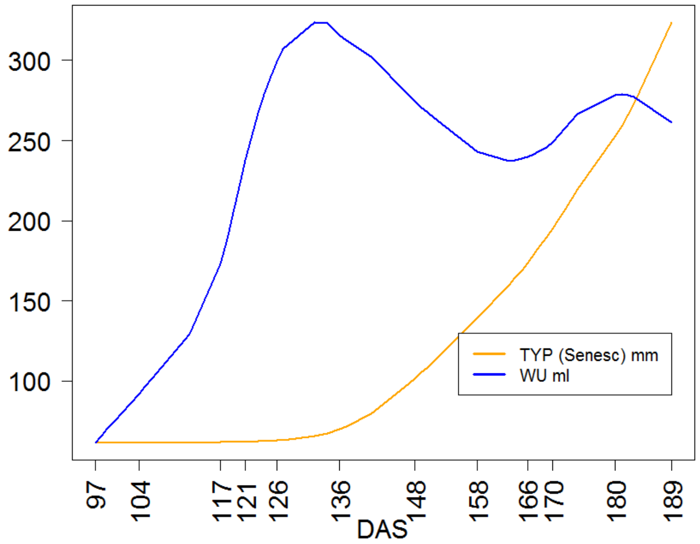

Figure S1 Average for all plants for Water use (WU) and whole plant senescence (TYP) plotted against days from sowing (DAS).

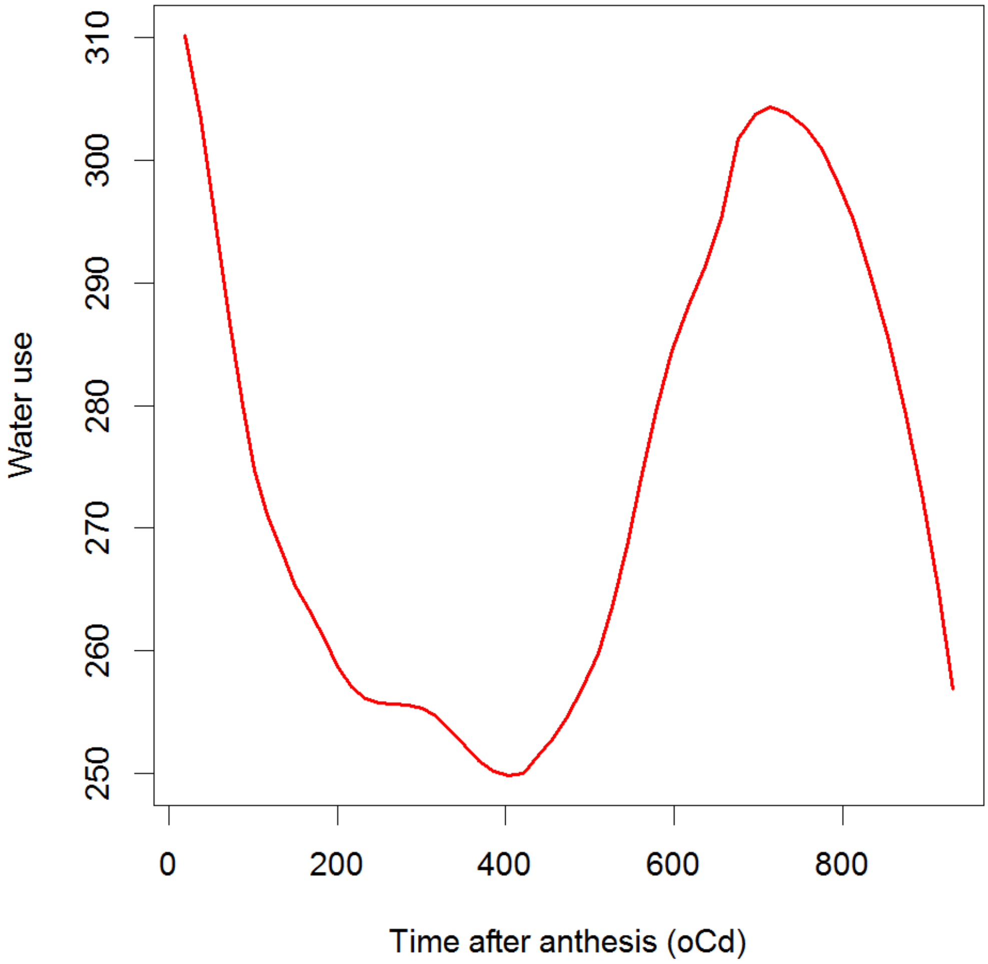

Figure S2. Average for all plants for Time after anthesis (ºCd) against water use (ml)

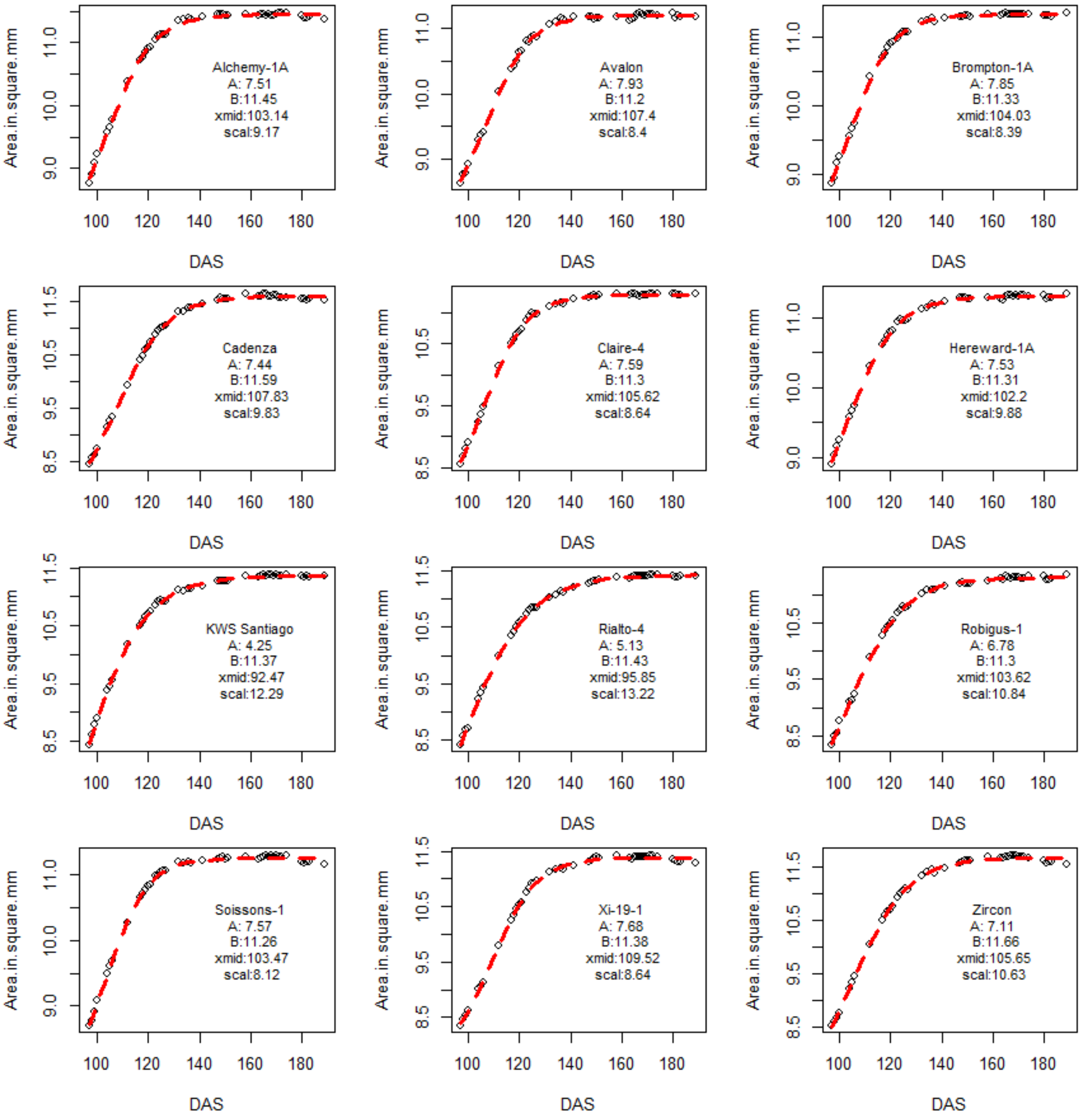

Figure S3 4-parameter Logistic curves fitted to plant Area from MAGIC parents

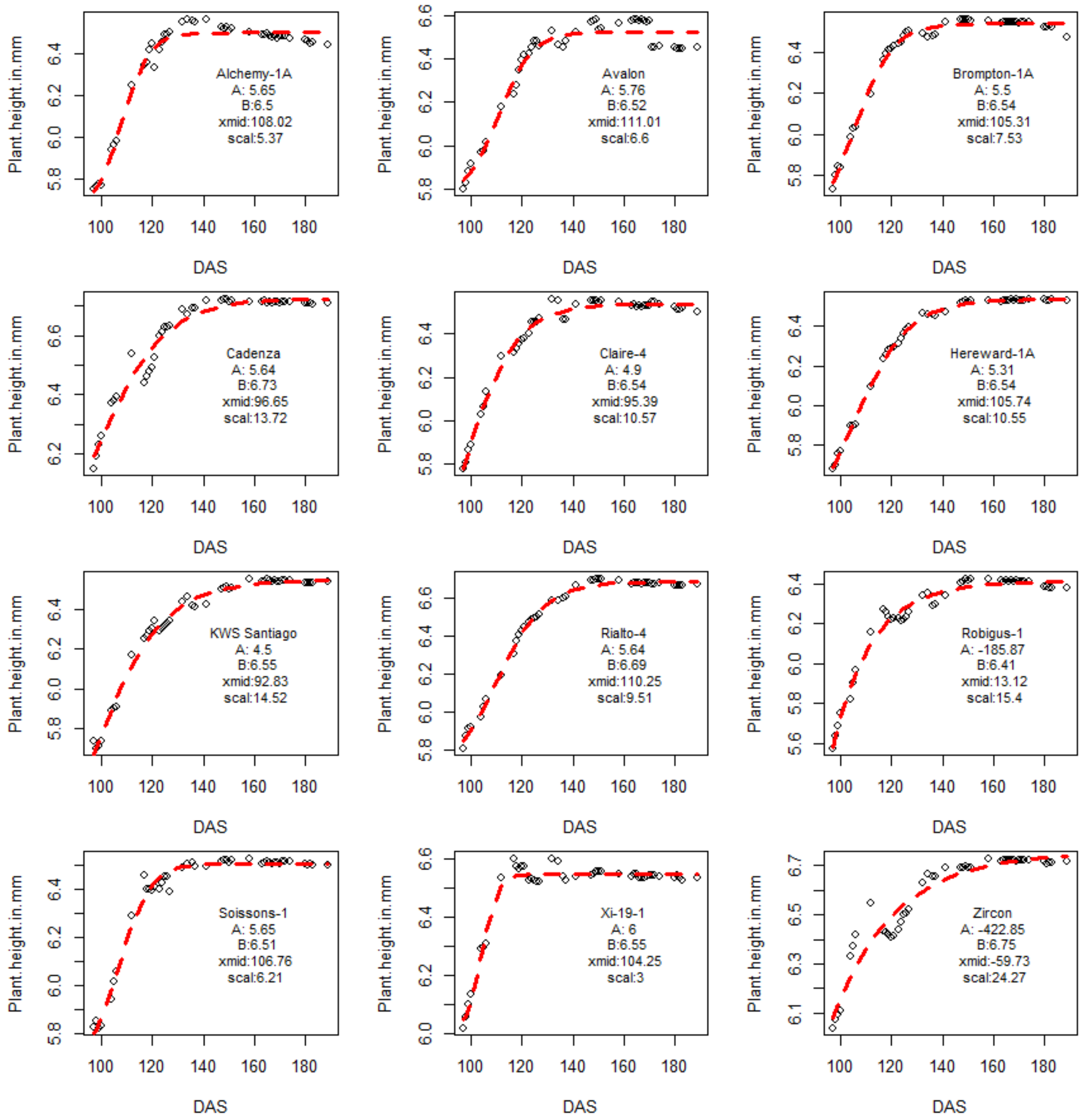

Figure S4 4-parameter Logistic curves fitted to plant Height from MAGIC parents.

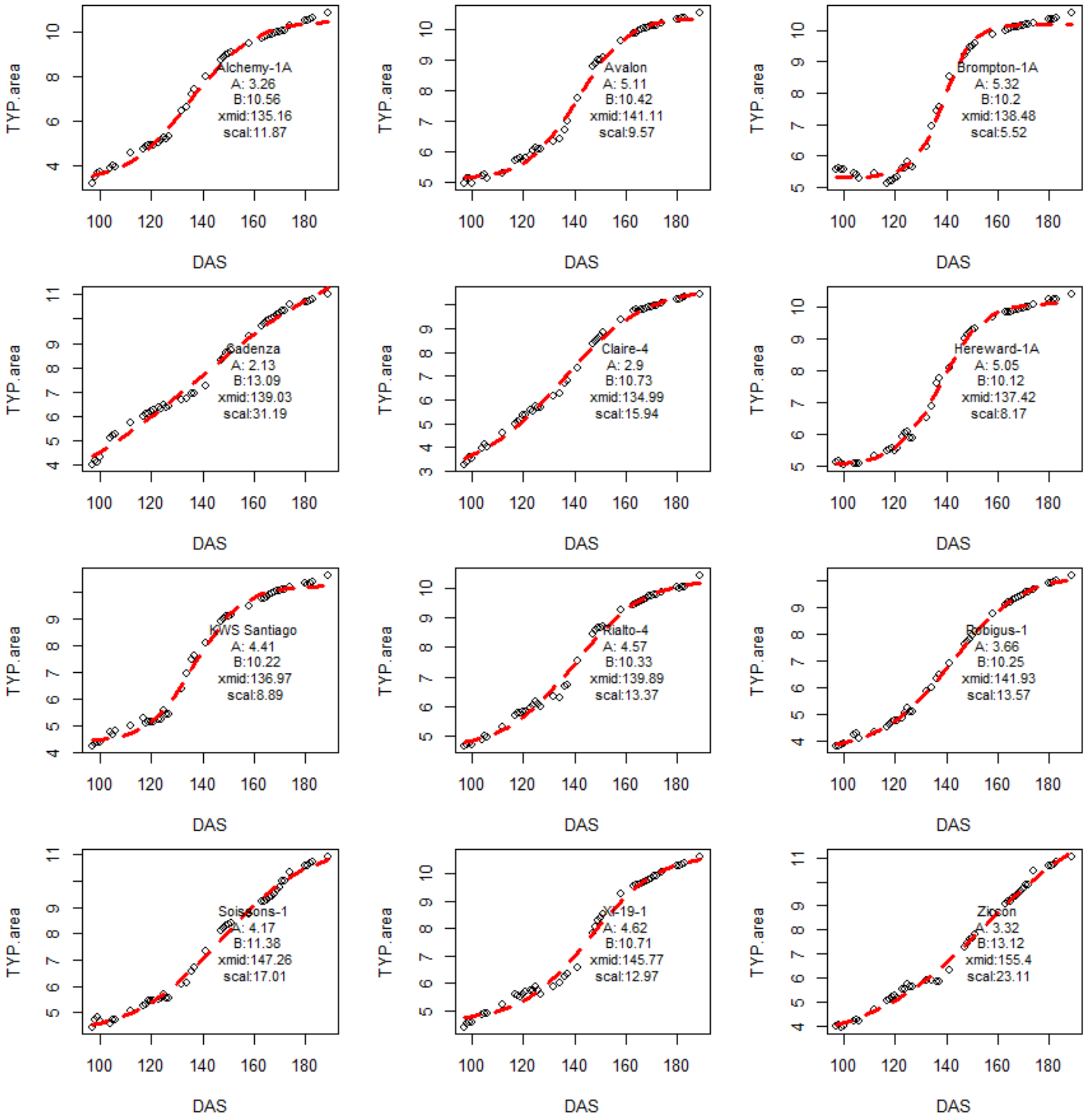

Figure S5 4-parameter logistic curves fitted to log TYP.area from MAGIC parents.

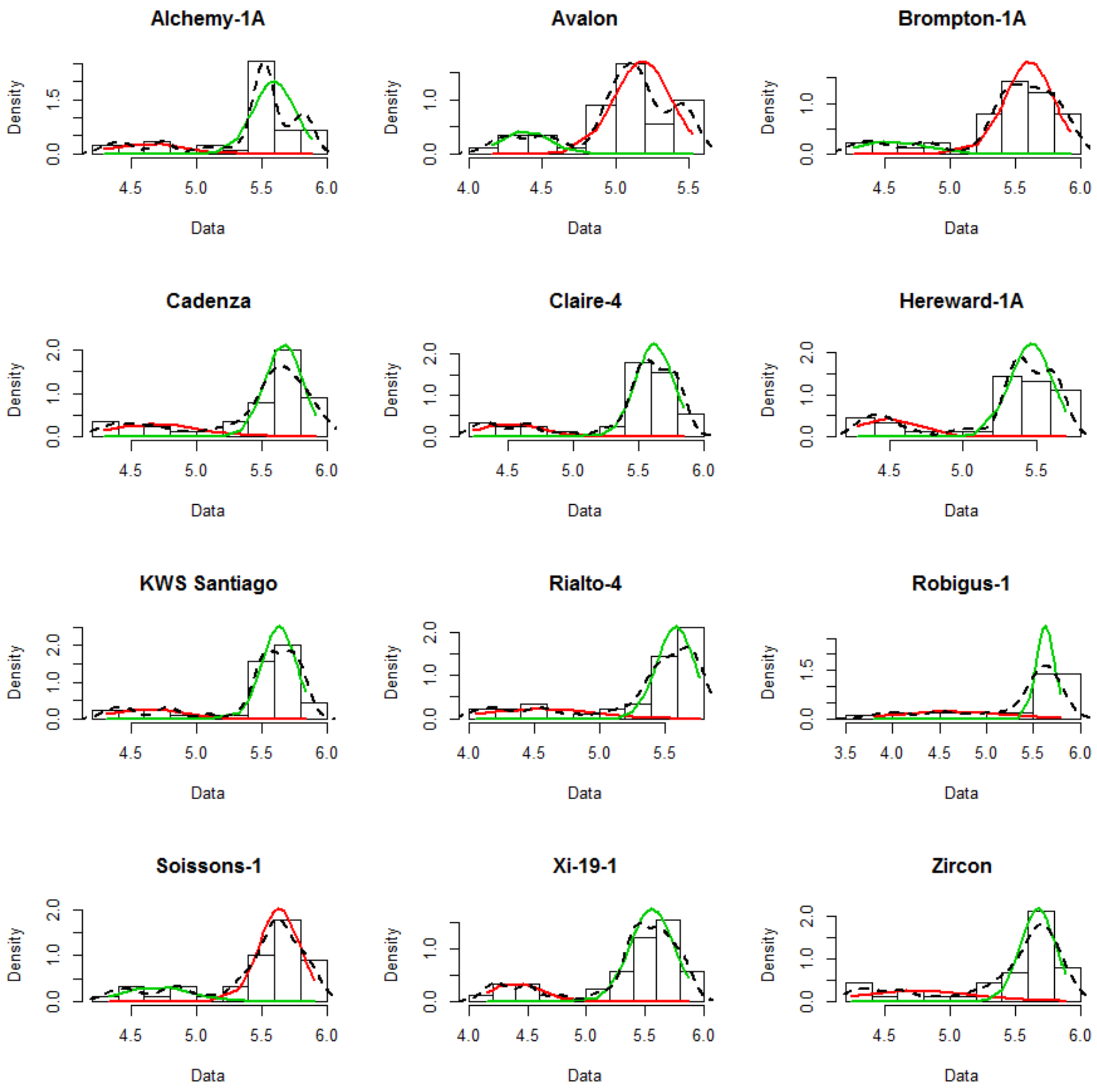

Figure S6 Histogram of data with m = 2 (two modes) fitted Gaussian components densities of $N(\hat{\mu}_j, \hat{\sigma}_j^2)$ each scaled by the corresponding λ_j, superimposed. Bi-modal growth curves were fitted to log water amount from MAGIC parents. Solid red and red lines are the fitted Gaussian components. Tallest mode (μ_1) correspond to the range of time when the average plant needed more water. Lowest mode (μ_2) corresponds to the range of time when the average increase water uptake after reaching a plateau.

Table S1 Statistical results from the analysis of growth parameters from RILs and for Area. H2 is the heritability for each trait *A, B, C* and *D*

	A	B	C	D
**genotype**	3.618	0.055	16.628	2.460
**Rep**	0.000	0.000	0.000	0.001
**Res**	9.388	0.012	44.739	2.579
**H2**	0.278	0.827	0.271	0.488

Table S2 covariance and correlation for genotype and residuals for Area, '-' indicates same parameter

genetic	A	B	C	D	environment	A	B	C	D
**correlation**					**correlation**				
**A**	-	-0.11	0.97	-0.77	**A**	-	0	0.91	-0.59
**B**	-	-	-0.2	0.35	**B**	-	-	0.08	0.28
**C**	-	-	-	-0.85	**C**	-	-	-	-0.52
**D**	-	-	-	-	**D**	-	-	-	-

Table S3 Statistical results from the analysis of growth parameters from RILs and for Height. H2 is the heritability for each trait *A, B, C* and *D*

	A	B	C	D
**genotype**	1.263	0.022	52.006	10.804
**rep**	0	0	0	0
**res**	4.754	0.004	97.968	9.557
**H2**	0.21	0.861	0.347	0.531

Table S4 covariance and correlation for genotype and residuals for Height, '-' indicates same parameter

genetic	A	B	C	D	environment	A	B	C	D
**correlation**					**correlation**				
**A**	-	-0.3	0.97	-0.82	**A**	-	-0.22	0.84	-0.47
**B**	-	-	-0.35	0.51	**B**	-	-	-0.21	0.41
**C**	-	-	-	-0.74	**C**	-	-	-	-0.68
**D**	-	-	-	-	**D**	-	-	-	-

Table S5 Statistical results from the analysis of growth parameters from RILs and for senescence (TYP). H2 is the heritability for each trait *A, B, C* and *D*

	A	B	C	D
**genotype**	0.502	0.091	19.266	8.331
**rep**	0.003	0.000	0.394	0.000
**res**	1.104	0.293	35.702	23.187
**H2**	0.313	0.237	0.350	0.264

Table S6 covariance and correlation for genotype and residuals for Senescence (TYP), '-' indicates same parameter

genetic	A	B	C	D	environment	A	B	C	D
**correlation**					**correlation**				
**A**	-	-0.42	0.11	-0.62	**A**	-	-0.63	0.34	-0.79
**B**	-	-	0.38	0.44	**B**	-	-	0.14	0.87
**C**	-	-	-	0.48	**C**	-	-	-	0.09
**D**	-	-	-	-	**D**	-	-	-	-

Table S7 Statistical results from the analysis of growth parameters from RILs and for water use.

	μ1	λ1	σ1	μ2	σ2
**genotype**	0.221	0.000	0.153	0.157	0.075
**rep**	0.032	0.000	0.249	0.000	0.117
**res**	0.764	1.000	0.723	0.843	0.867
**H2**	0.224	0.000	0.175	0.157	0.080

Table S8 covariance and correlation for genotype and residuals for water use, '-' indicates same parameter and 'NA' indicates missing.

genetic	μ1	λ1	σ1	μ2	σ2	environment	μ1	λ1	σ1	μ2	σ2
**correlation**						**correlation**					
**μ1**	-	NA	-1.02	0.22	0.21	**μ1**	-	0.72	-0.77	-0.46	0.19
**λ1**	-	-	NA	NA	NA	**λ1**	-	-	-0.31	-0.84	0.11
**σ1**	-	-	-	0	-0.25	**σ1**	-	-	-	0.27	-0.18
**μ2**	-	-	-	-	-1.07	**μ2**	-	-	-	-	-0.14
**σ2**	-	-	-	-	-	**σ2**	-	-	-	-	-

Table 9. QTLs mapped for growth curve parameters corresponding to (Area, Height, Senescence and water use). cM is the marker position. h2 is heritability. logP is -log10 at the QTL peak. Chr is the chromosome. P-value is the genome wise P-value for the QTL based on permutations. Alchemy, Brompton, Claire, Hereward, Rialto, Robigus, Soissons and Xi-19 indicate the estimated founder QTL effects as produced by HAPPY. Last two columns show 90% confidence interval (CI) for the QTL based on permutations. These values are island intervals, they are the segments exceeding the genome-wide significance level.

Trait	DAS	peak.SNP	chr	cM	Alchemy	Brompton	Claire	Hereward	Rialto	Robigus	Soissons	Xi19	h2	logP	P-value	lower	upper
Area	136	RAC875_rep_c105718_585	chr4D	40.11	69244.19	70726.64	68961.25	71277.8	64324.87	88639.24	82420.8	68635.23	0.26	5.03	0	1100001	2100001
Area	148	RAC875_rep_c105718_585	chr4D	40.11	79011.69	80634.7	79132.84	79329.72	72228.77	104657.47	95155	78610.13	0.30	6.13	0	1100001	2200001
Area	158	RAC875_rep_c105718_585	chr4D	40.11	83601.38	84625.25	83505.17	82474.3	74737.2	110709.22	99681.57	81966.03	0.32	6.84	0	1100001	2300001
Area	166	RAC875_rep_c105718_585	chr4D	40.11	85421.27	86303.78	84829.58	85063.95	76910.64	115352.03	101544.8	84263.48	0.33	7.24	0	1100001	2300001
Area	170	RAC875_rep_c105718_585	chr4D	40.11	84679.14	86124.21	85332.53	84096.08	76355.65	114026.02	100925.26	84268.74	0.32	7.01	0	1100001	2200001
Area	180	RAC875_rep_c105718_585	chr4D	40.11	84520.25	83636.76	84650.83	83425.9	75116.47	113026.45	97528.08	81995.76	0.32	6.98	0	1100001	2300001
Area	189	RAC875_rep_c105718_585	chr4D	40.11	82732.5	82136.26	83503.19	81775.89	72993.25	111018.8	94719.31	81649.07	0.32	6.69	0	1100001	2300001
Height	104	IAAV1650	chr5A	227.15	359.21	351.54	372.03	365.19	366.47	370.33	365.25	483.14	0.38	8.5	0	62600001	78600001
Height	117	Kukri_rep_c68594_530	chr4D	24.93	549.21	552.46	547.53	529.87	546.62	605.29	602.12	538.92	0.32	6.9	0	1000001	2100001
Height	117	Tdurum_contig42083_1539	chr4D	3.09	560.86	577.26	562.7	546.19	547.5	582.53	573.2	580.93	0.28	5.52	0	100001	200001
Height	121	RAC875_c1673_193	chr4D	32.24	587.3	595.07	586.07	585.94	586.81	656.97	665.65	574.99	0.25	4.71	0.01	1500001	2100001
Height	121	RAC875_c6922_291	chr4D	26.97	599.51	601.28	600.33	593.75	600.11	649.53	641.04	580.64	0.30	6.2	0	1000001	1300001
Height	121	Tdurum_contig42083_1539	chr4D	3.09	610.88	620.54	608.56	609	594.23	629.3	621.7	627.37	0.23	4.09	0.04	100001	200001
Height	126	RAC875_c6922_291	chr4D	26.97	635.91	639.62	634.65	629.29	636.49	688.47	678.17	617	0.34	7.59	0	1000001	2100001
Height	126	Tdurum_contig42083_1539	chr4D	3.09	647.73	663.12	650.67	639.26	638.69	667.54	659.18	647.46	0.22	3.83	0.05	100001	200001
Height	136	RAC875_c1673_193	chr4D	32.24	665.19	672.13	664.86	668.97	670.64	748.67	752.33	655.72	0.32	6.84	0	1000001	2200001
Height	148	RAC875_rep_c105718_585	chr4D	40.11	690.27	741	689.67	695.06	699.69	826.91	798.72	687.68	0.41	10.12	0	1000001	2800001
Height	158	RAC875_c1673_193	chr4D	32.24	709.59	718.92	705.97	713.13	703.46	837.5	820.89	691.51	0.42	10.47	0	1000001	2800001
Height	166	RAC875_c1673_193	chr4D	32.24	706.59	716.82	715.03	709.11	708.93	855.32	839.75	695.3	0.40	9.62	0	1000001	2500001
Height	170	RAC875_c1673_193	chr4D	32.24	711.2	723.01	711.97	709.62	708.92	860.57	835.8	693.25	0.40	9.75	0	1000001	2500001
Height	180	RAC875_c1673_193	chr4D	32.24	705.56	713.01	710.01	708.75	700.14	856.26	831.98	691.63	0.40	9.77	0	1000001	2500001
Height	189	RAC875_c1673_193	chr4D	32.24	703.12	712.39	697.06	704.36	697.64	850	831.53	681.1	0.41	10.02	0	1000001	2400001
Height	97	BS00011360_51	chr5A	229.67	290.19	287.85	302.31	289.38	299.26	296.85	299.37	374.83	0.31	6.16	0	62700001	78500001
Height	97	wsnp_Ex_c113235_94249366	chr5B	61.66	314.61	314.17	311.8	311.75	310.42	283.53	300.78	262.24	0.23	3.71	0.08	47700001	47900001

Height	97	wsnp_Ra_c44756_51084202	chr5B	60.15	315.15	311.85	311.73	310.33	310.27	287.56	305.17	262.71	0.23	3.61	0.1	47500001	47600001
TYArea	104	RFL_Contig167_504	chr3B	163.5	124.38	79.85	73.95	49.3	71.91	82.69	75.66	72.69	0.26	4.49	0.07	92100001	92600001
TYArea	148	RHT2	chr4D	34.28	6133.67	5836.9	5998.87	6162.2	5701.7	8185	7987.47	5823.81	0.23	3.99	0.08	1600001	1700001
TYArea	166	Kukri_c27309_590	chr2D	55.4	20203.98	18583.62	20025.34	20084.7	18496.39	18941.17	13170.77	19521.4	0.25	4.2	0.03	13800001	13900001
TYArea	170	RHT2	chr4D	34.28	19687.5	19740.16	19759.82	19769.83	19205.28	24537.55	23453.34	19127.6	0.22	3.93	0.05	1600001	1700001
TYArea	170	Kukri_c27309_590	chr2D	55.4	22672.1	21068.55	22795.22	22760.73	21282.04	21611.08	15391.02	21840.67	0.24	3.99	0.05	13800001	13900001
TYArea	180	RAC875_c1673_193	chr4D	32.24	27708.2	27568.07	27379.38	27410.43	26809.1	38035.26	38044.2	25882.88	0.26	4.9	0.01	1300001	2100001
TYArea	189	RAC875_c1673_193	chr4D	32.24	36642.01	36791.6	36739.89	37302.61	35809.98	52920.44	51199.04	35243	0.33	7.04	0	1100001	2100001
WA	148	GENE_0137_147	chr2D	53.36	301.65	289.39	294.24	298.44	297.12	302.92	256.26	302.82	0.24	3.86	0.06	12500001	13700001
WA	158	GENE_0137_147	chr2D	53.36	242.79	233.5	237.44	241.51	240.05	244.43	203.84	241.79	0.25	4.19	0.02	12500001	13700001
WA	166	BS00069245_51	chr5A	310.54	222.36	213.22	221.95	222.51	225.45	204.56	271.01	208.91	0.23	3.85	0.05	93400001	94100001
WA	166	Excalibur_c27357_146	chr5A	313.57	223.86	215.36	222.67	224.99	226.86	205.61	268.6	207.08	0.23	3.63	0.08	94800001	95000001
WA	170	BS00069245_51	chr5A	310.54	235.31	226.8	234.87	234.59	246.89	223.1	282.97	226.23	0.23	3.61	0.1	93500001	94100001
WA	189	Kukri_c27309_590	chr2D	55.4	267.54	270.93	262.73	261.49	266.55	265.14	219.79	271.34	0.23	3.61	0.09	13800001	13900001
At		BS00004376_51	chr7B	259.62	3.78	3.5	3.66	3.96	3.51	2.44	3.26	3.93	0.24	3.86	0.08	75400001	76000001
At		BS00087197_51	chr7B	253.51	3.82	3.6	3.58	4.05	3.59	2.78	3.05	3.82	0.25	4.25	0.05	74600001	74700001
At		Tdurum_contig52096_270	chr7B	255.55	3.78	3.56	3.59	4.02	3.56	2.67	3.24	3.83	0.24	3.79	0.1	74800001	74900001
B		BS00022276_51	chr2D	53.36	11.4	11.38	11.33	11.32	11.35	11.41	11.2	11.43	0.23	3.84	0.05	13700001	13900001
B		RAC875_rep_c105718_585	chr4D	40.11	11.29	11.3	11.3	11.31	11.2	11.63	11.48	11.31	0.31	6.58	0	1100001	2200001
Bh		RAC875_c1673_193	chr4D	32.24	6.53	6.54	6.52	6.55	6.54	6.75	6.68	6.52	0.38	9.09	0	1100001	2500001
lambda1		GENE_4528_28	chr7B	216.43	0.22	0.29	0.22	0.22	0.28	0.22	0.25	0.32	0.27	4.75	0.1	63800001	64600001
mu2		BS00022810_51	chr7B	216.43	5.53	5.39	5.58	5.56	5.41	5.59	5.4	5.29	0.30	5.4	0.06	62800001	64900001
mu2		RAC875_c10372_542	chr7B	227.25	5.52	5.39	5.54	5.59	5.38	5.56	5.43	5.31	0.28	4.94	0.09	68800001	68900001
mu2		Tdurum_contig44876_1570	chr7B	208.29	5.53	5.4	5.58	5.51	5.42	5.58	5.4	5.32	0.28	4.89	0.1	62600001	62700001

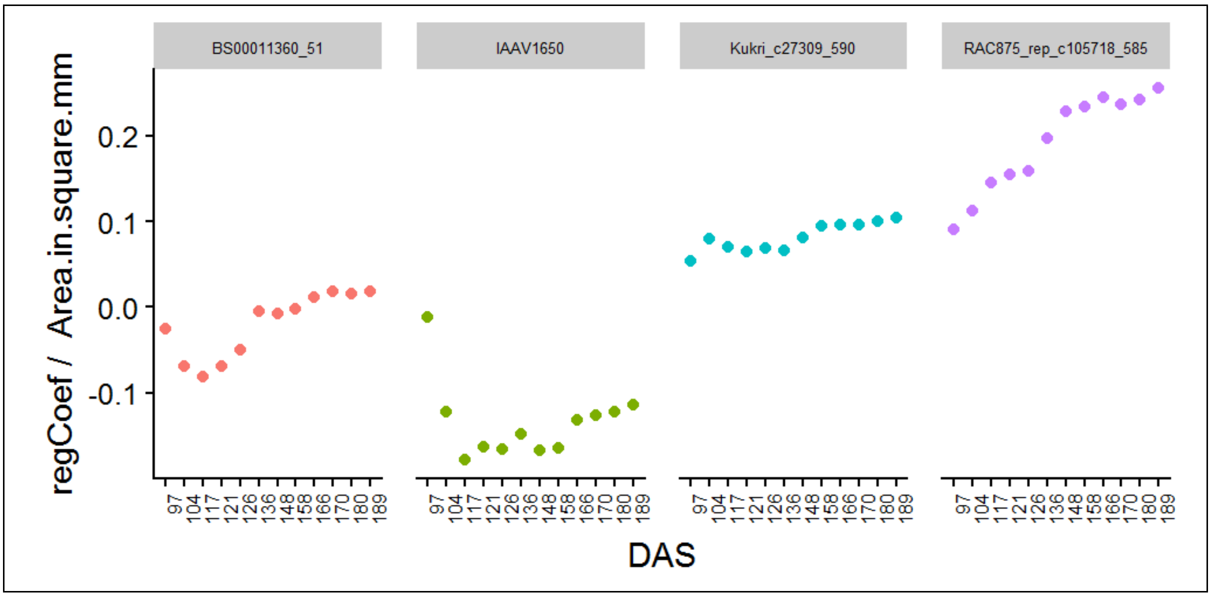

58 **Figure S7**. Marker effect on plant Area

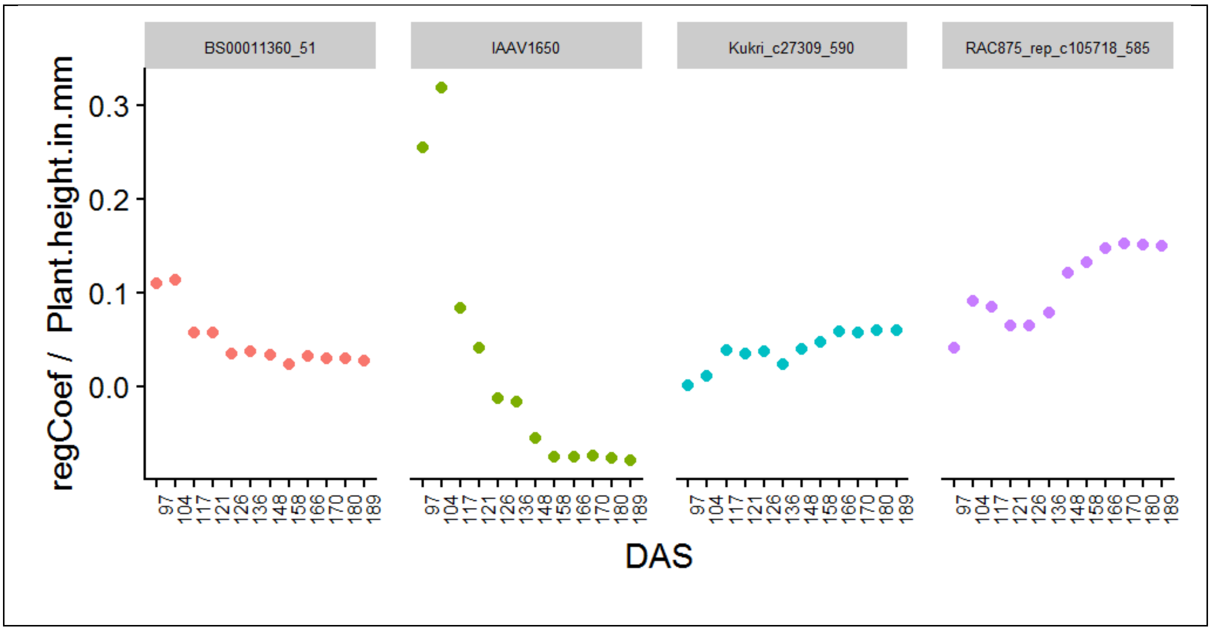

59 **Figure S8** Marker effect on plant Height

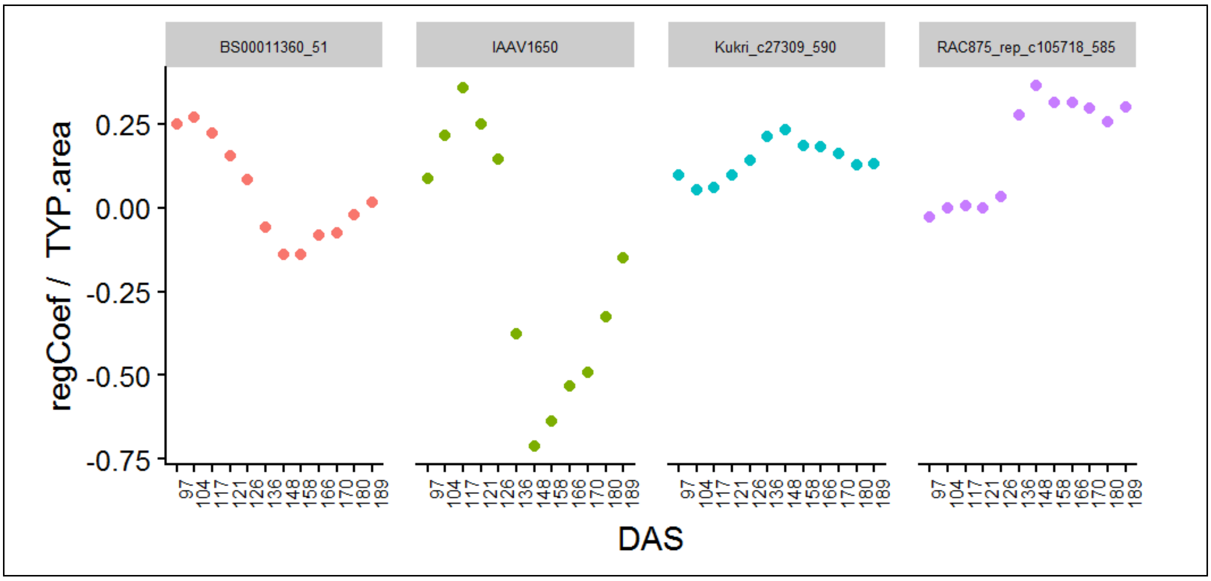

Figure S9 Marker effect on plant Senescence

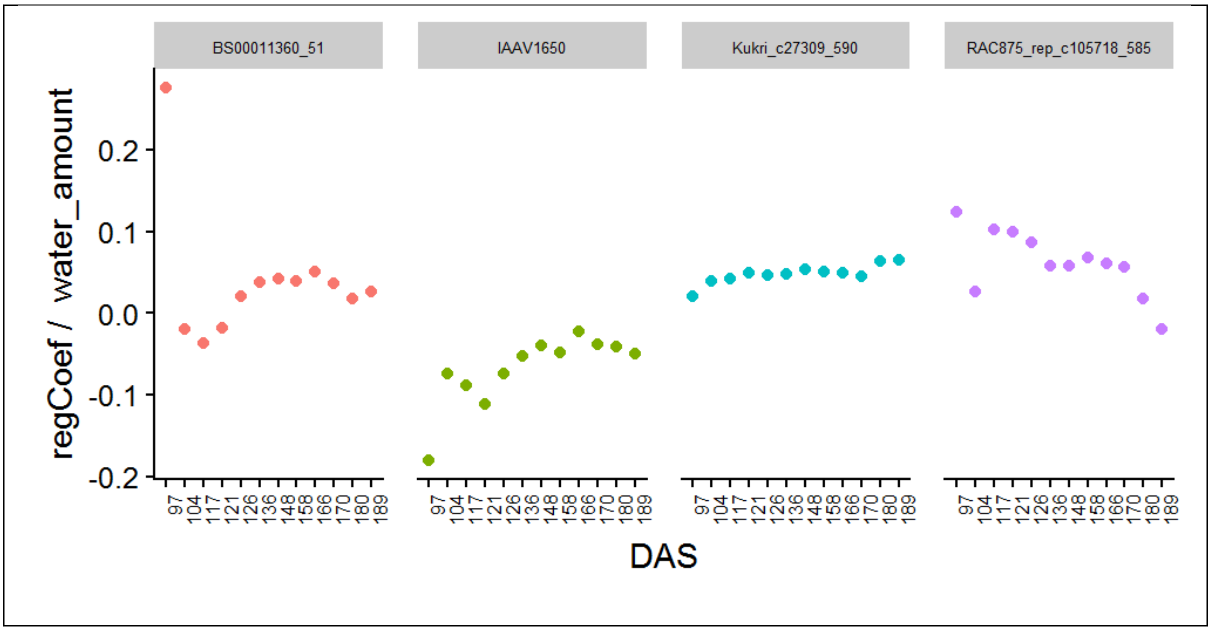

Figure S10 Marker effect on plant water use

63

No	m1	m2	m3	m4	97 DAS	148 DAS	180 DAS
1	2	2	2	2	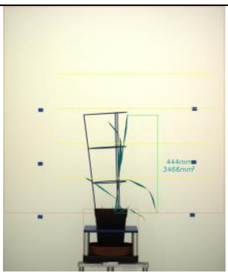	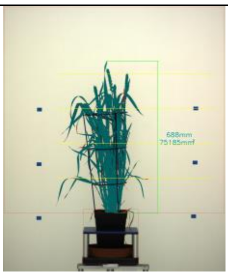	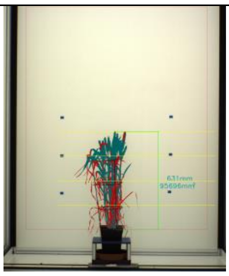
2	0	2	2	2	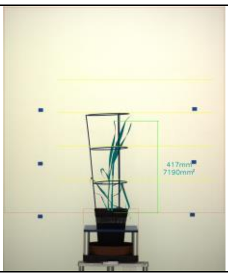	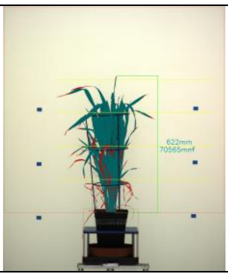	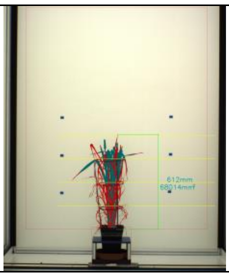
3	2	0	2	2			
4	0	0	2	2			
5	2	2	0	2	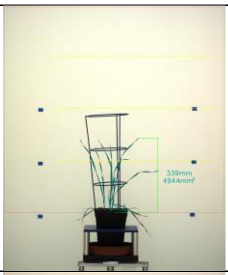	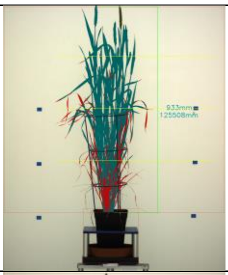	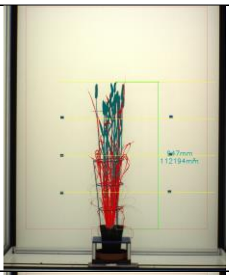
6	0	2	0	2	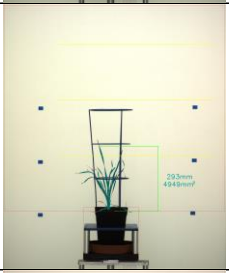	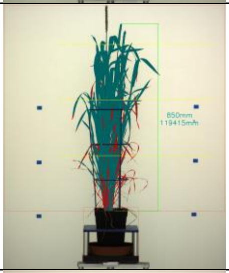	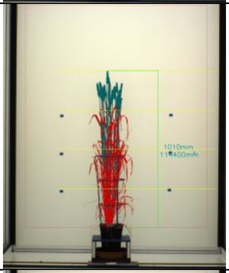
7	2	0	0	2	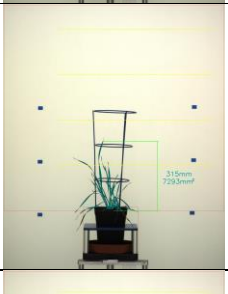	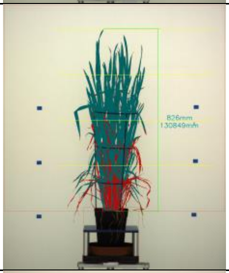	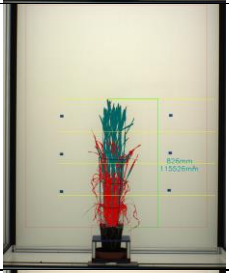
8	0	0	0	2	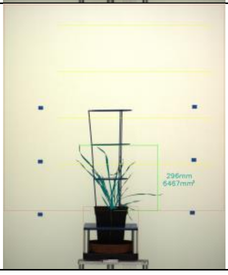	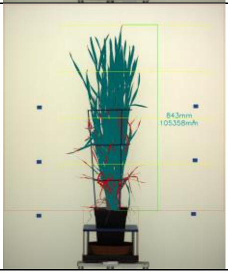	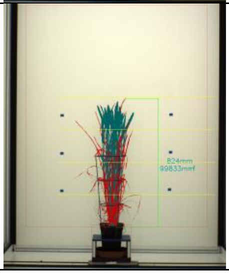

9	2	2	2	0	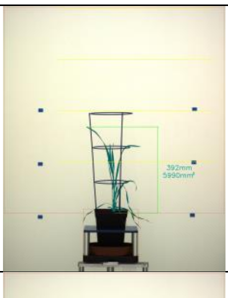	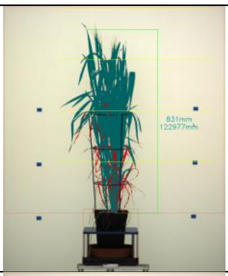	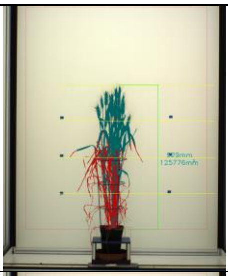
10	0	2	2	0	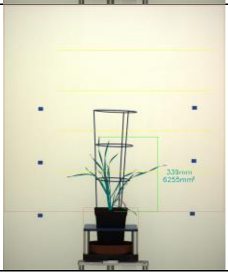	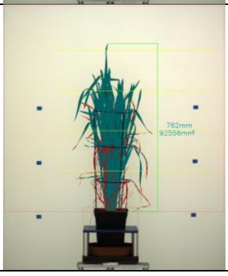	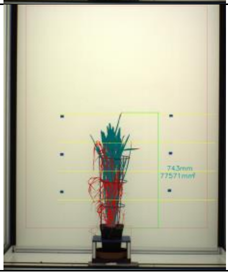
11	2	0	2	0			
12	0	0	2	0	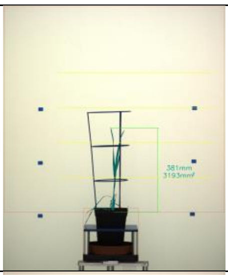	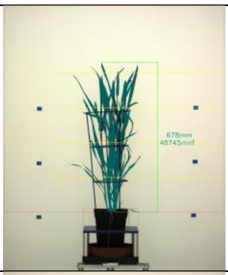	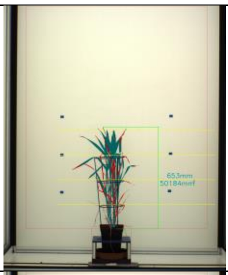
13	2	2	0	0	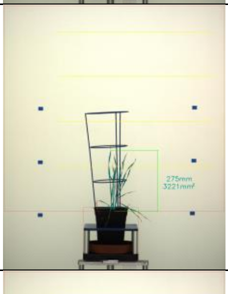	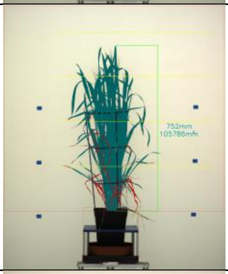	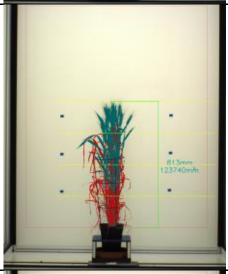
14	0	2	0	0	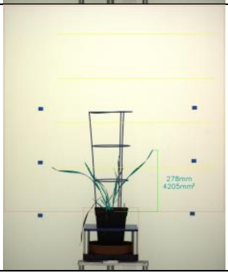	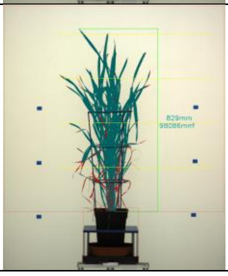	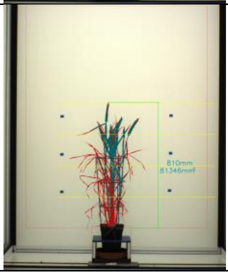
15	2	0	0	0			
16	0	0	0	0	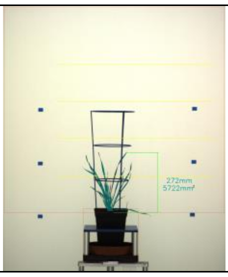	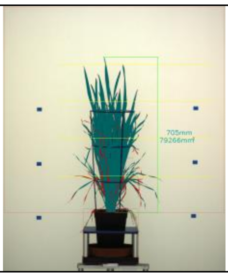	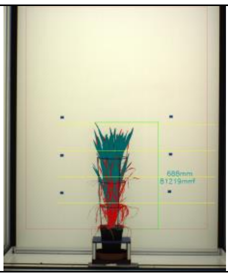

Figure S11. Comparison of phenotypes and genotypes for four markers m1=RAC875_rep_c105718_585, m2=BS00011360_51, m3= IAAV1650 and m4 =

Kukri_c27309_590. Green area is the plant are coloured in green and yellow areas in red. The zoom in the camera was decreased at 180 DAS to allow the whole plant to fit in the image.
